# Machine learning reveals immediate disruption in mosquito flight when exposed to Olyset nets

**DOI:** 10.1016/j.crpvbd.2025.100273

**Published:** 2025-05-22

**Authors:** Yasser M. Qureshi, Vitaly Voloshin, Amy Guy, Hilary Ranson, Philip J. McCall, James A. Covington, Catherine E. Towers, David P. Towers

**Affiliations:** a School of Engineering, University of Warwick, Coventry, CV4 7AL, UK; b School of Biological and Behavioural Sciences, Queen Mary University of London, E1 4NS, UK; c Vector Biology Department, Liverpool School of Tropical Medicine, Pembroke Place, Liverpool, L3 5QA, UK

**Keywords:** Insecticide-treated nets, Insecticide resistance, Mosquito behaviour, Machine learning, Trajectories, Malaria control, *Anopheles*

## Abstract

Insecticide-treated nets (ITNs) remain a critical intervention in controlling malaria transmission, yet the behavioural adaptations of mosquitoes in response to these interventions are not fully understood. This study examined the flight behaviour of insecticide-resistant (IR) and insecticide-susceptible (IS) *Anopheles gambiae* strains around an Olyset net (OL), a permethrin-impregnated ITN, *versus* an untreated net (UT). Using machine learning (ML) models, we classified mosquito flight trajectories with high balanced accuracy (0.838) and ROC AUC (0.925). Contrary to assumptions that behavioural changes at OL would intensify over time, our findings show an immediate onset of convoluted, erratic flight paths for both IR and IS mosquitoes around the treated net. SHAP analysis identified three key predictive features of OL exposure: frequency of zero-crossings in flight angle change; first quartile of flight angle change; and zero-crossings in horizontal velocity. These suggest disruptive flight patterns, indicating insecticidal irritancy. While IS mosquitoes displayed rapid, disordered trajectories and mostly died within 30 min, IR mosquitoes persisted throughout the 2-h experiments but exhibited similarly disturbed behaviour, suggesting resistance does not fully mitigate disruption. Our findings challenge literature suggesting permethrin’s repellency in solution form, instead supporting an irritant or contact-driven effect when incorporated into net fibres. This study highlights the value of ML-based trajectory analysis for understanding mosquito behaviour, refining ITN configurations and evaluating novel active ingredients aimed at disrupting mosquito flight behaviour. Future work should extend these methods to other ITNs to further illuminate the complex interplay between mosquito behaviour and insecticidal intervention.

## Highlights

• ITNs are vital in malaria control but need deeper insight into mosquito behaviour.
• ML classifies mosquito flight near treated/untreated nets with ROC AUC over 0.93.
• Insecticide disrupts flight instantly, suggesting irritancy, not repellency.
• Resistant and susceptible mosquitoes exhibit similarly chaotic flight behaviours.
• Key trajectory features predict exposure, revealing erratic moves and disorientation.

## Graphical abstract

**Figure:**
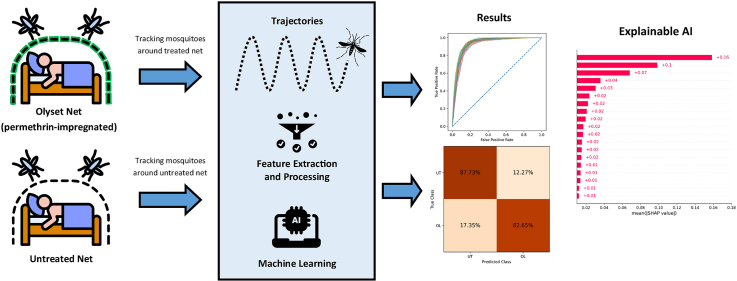

## 1 Introduction

Mosquito-borne diseases are among the leading causes of human mortality, with over one million deaths per annum worldwide, and they are a barrier to development in countries where they are endemic (Chilakam et al., 2023). Control of mosquitoes with insecticide-based interventions has been a critical part of the global strategy to manage mosquito-borne diseases (Liu, 2015). Pyrethroids are among the most widely used insecticides, delivered historically by indoor residual spraying walls and today primarily as insecticide-treated nets (ITNs), they are highly effective in malaria vector control. However, prolonged, widespread use led to the appearance of resistance which spread rapidly in mosquito populations across Africa following the rollout of ITNs in the first years of the 21st Century. In that timeframe, the malaria burden in Africa had been reduced by 50%, most of which was attributed to ITNs (Bhatt et al., 2015). By 2015, the pace of progress had declined considerably, and malaria cases have plateaued in recent years (Ranson and Lissenden, 2016). An understanding of the mechanisms governing the development of insecticide resistance has led to new and more effective strategies to control resistant mosquitoes, including next-generation ITNs. Whilst much is known about the genetic mechanisms of insecticide resistance, far less attention has been paid to understanding the full range of the impacts of pyrethroid exposure on mosquitoes and malaria transmission (Parker et al., 2015; Jones et al., 2021; Hall et al., 2022).

The Olyset net, in 2001, was the first ITN to be validated under the WHO Pesticide Evaluation Scheme (WHOPES) and today it is used in over 80 countries, protecting nearly 800 million people (https://sumitomo-chem.com.sg/olyset-net/). Despite widespread pyrethroid resistance, Olyset nets can still impact resistant mosquitoes by reducing their willingness to feed, acting as a barrier, and/or reducing their lifespan (Ngongang-Yipmo et al., 2022; Gleave et al., 2023). Due to their lower cost, they continue to be used in areas where insecticide resistance levels are low despite the widespread availability of next-generation of dual-active ingredient nets (Qureshi, 2018; Carnevale and Gay, 2019).

Recent studies have explored behavioural responses of insecticide-resistant mosquitoes on a number of ITNs, including Olyset and some dual-active ingredient nets (Gleave et al., 2023). By tracking various mosquito strains around different human-baited ITNs for two hours using an infrared video system, it was concluded that were no major differences in the duration or character of net contact, repellency or irritability of the different nets, or of the consequences of exposure on longevity between pyrethroid-resistant and susceptible mosquitoes when exposed to ITNs. The analysis primarily used statistical methods to examine parameters, such as net contact duration, mosquito activity decay, and feeding inhibition. Here, we report on the application of machine learning (ML) models to classify mosquito behaviours based on similar data collected from Olyset and untreated nets. By leveraging explainable AI (XAI) techniques, we have revealed further insights into mosquito movement patterns and on subtle behavioural differences between resistant and susceptible strains that were not detected through traditional statistical methods.

ML models can identify flight characteristics that distinguish groups of mosquitoes. For instance, Qureshi et al. (2023) were able to identify behaviours between sexes of mosquitoes with an average balanced accuracy and ROC AUC score of 0.645 and 0.684, which represented the first work to use ML models to explore mosquito flight behaviour *via* trajectories. Similar work (Qureshi et al., 2025) was also performed to differentiate between insecticide-susceptible (IS) and insecticide-resistant (IR) behaviours at an untreated net, with the best model returning an average balanced accuracy of 0.743 and a ROC AUC score of 0.813. Furthermore, the model was then explained using SHapley Additive exPlanations (SHAP) values (Lundberg and Lee, 2017) revealed that IR mosquitoes tend to exhibit more directed flight paths with some jerky motion, possibly allowing the mosquito to sample attractants and maintain flight towards the highest concentration of these cues and hence a potential blood meal. These metrics were deliberately chosen for their suitability in handling imbalanced data and offer strong evidence that the models were able to extract meaningful behavioural patterns - though, as with all such analyses, interpretations should consider the nature of the underlying data.

In this work, we present the first evidence of the behavioural impact of ITNs on mosquito flight using an established ML approach. The study utilised trajectory data of mosquitoes flying around an untreated baited bednet and a treated bednet (Olyset) in order to identify behaviours resulting from exposure to the insecticide. Features of flight were extracted from the trajectories and provided to a ML model to attempt to distinguish between the two net types. Then, SHAP’s analysis was used to explain the model predictions.

## 2 Materials and methods

### 2.1 Dataset description

The dataset used was generated within laboratory experiments at the Liverpool School of Tropical Medicine (LSTM), UK (Gleave et al., 2023). Within each experiment, unfed adult female mosquitoes were tracked around a human-baited bednet. A total of 17 human volunteers were used as the bait to reflect natural host variability; volunteers were randomly assigned to net type and strain. In 17 of the experiments, the bednet was untreated (i.e. no presence of insecticide), in 23 of the experiments, the bednet was a standard pyrethroid-only net (Olyset Net™). Olyset nets are made from polyethylene fibres with permethrin (2%) incorporated directly into the material during manufacturing, rather than being surface-coated like conventional ITNs. The manufacturer states that this allows the insecticide to gradually migrate to the surface over time, and that Olyset nets must be activated before first use by heating, after which time they will provide effective protection for a few years (Ito and Okuno, 2006).

The two bednets (Olyset and untreated control) were matched as closely as possible in terms of size, shape, and positioning within the experimental setup. Both net types were adjusted to fit snugly on an identical frame (90 cm × 180 cm roof size; 45 cm high at the front and 75 cm at the rear), with excess netting trimmed to ensure uniform structure and maximise visibility of mosquito activity. Nets were suspended using the same carbon rod frame, and both setups were tilted in the same manner to facilitate observation of the roof area. The only visible difference between the nets was their colour: the untreated control net was white, while the Olyset net was blue. We acknowledge that this colour difference may have introduced a minor visual cue, although care was taken to control for all other variables.

Four *Anopheles gambaie* (*sensu lato*) mosquito strains were used, two insecticide-susceptible (Kisumu and N’Gousso) and two insecticide-resistant (VK7 and Banfora). The Kisumu strain (*Anopheles gambiae* (*sensu stricto*)), first collected in Kenya in 1975, and the N’Gousso strain (*Anopheles coluzzii*), established in 2006 from Cameroon, are both insecticide-susceptible mosquito populations (Williams et al., 2019). In contrast, the VK7 and Banfora strains (both *Anopheles coluzzii*), derived from Burkina Faso in 2014 and 2015, exhibit high levels of pyrethroid resistance. This resistance is primarily driven by *kdr* mutations (995F in VK7 and a mix of 995F and 402L-1527 in Banfora) and metabolic adaptations, including elevated cytochrome P450 expression (Williams et al., 2019, 2022).

To preserve these resistance traits, insecticide-resistant strains were periodically exposed to 0.05% deltamethrin-treated papers every 3–5 generations, following the WHO susceptibility bioassay protocol (WHO, 2016). Annual bioassays were conducted to assess susceptibility to a range of insecticides, including pyrethroids, carbamates, and organophosphates. Molecular genotyping was also routinely performed to verify the presence of *kdr* (995F and, more recently, 402L alleles) mutations and elevated cytochrome P450 expression, ensuring the stability of resistance mechanisms over time. Further details on strain origins and maintenance protocols are available in Williams et al. (2019).

**Fig. 1:**
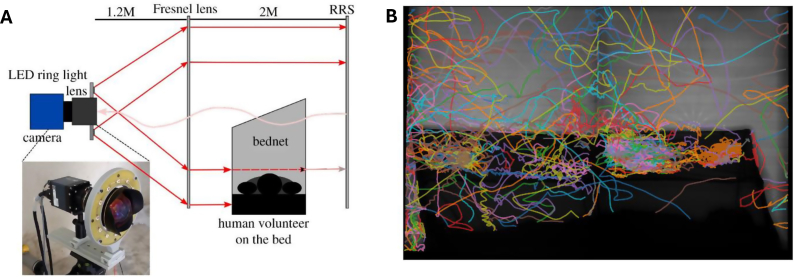
**A** Schematic of the experimental setup used for mosquito flight tracking, showing the camera and imaging lens surrounded by an LED infrared ring light, a Fresnel lens, and a retroreflective screen (RRS) to enhance flight trajectory capture. This schematic is adapted from (Qureshi et al., 2025), which itself was adapted from earlier work (Voloshin et al., 2020). **B** Example flight trajectories recorded during a Kisumu trial, with each coloured line representing the path of an individual mosquito over the first 15 min of the experiment, illustrating typical mosquito movement patterns around the bednet.

Experiments, referred to as trials, were conducted between June 2019 and February 2020 in a custom-built, climate-controlled free-flight testing room (7 × 4.8 m, 2.5 m high). Mosquitoes were reared under a reversed 12:12 h light/dark cycle to ensure that trials conducted in the afternoon coincided with their subjective ‘night’ phase, when host-seeking behaviour typically occurs. Each of the 40 trials involved the release and tracking of 25 individual mosquitoes over a 2-h period, resulting in a total of 1000 mosquitoes across all trials. Mosquitoes were tracked using paired recording systems, each comprising a 12 MPixel Ximea CB120RG-CM camera with a 14 mm focal length lens. Each camera was aligned with a Fresnel lens (1400 × 1050 mm, 3 mm thick, 1.2 m focal length; NTKJ Co., Ltd, Osaka, Japan) positioned approximately 1210 mm away. Retroreflective screens (2.88 m^2^, coated with high-gain sheeting) were placed behind the bednet to enhance light capture and improve contrast for video tracking (Voloshin et al., 2020). Infrared illumination (850 nm wavelength) enabled night-phase tracking without disturbing the mosquitoes, capturing trajectories at 50 frames per second. As such, the camera system’s field of view spans approximately 1.2 m^2^. Figure 1 illustrates the experimental setup and example flight trajectories obtained.

The system produced 2D telecentric data on mosquito flight patterns. Further details on the experimental setup can be found in Gleave et al. (2023), and trajectory extraction methods are described in Voloshin et al. (2020). Table 1 summarises the dataset, including a minimum track duration threshold of 1 s. Although mosquitoes fly in three dimensions, the 2D telecentric imaging system captures high-fidelity projections that effectively eliminate perspective distortion and have been shown to produce behavioural features comparable to those extracted from 3D data. Recent work demonstrated that 2D telecentric trajectories are well suited for machine learning applications, offering similar interpretability and classification performance to 3D tracking in the context of mosquito behaviour analysis (Qureshi et al., 2024).

**Table 1:** Information on the trajectories of each mosquito strain.

Strain	Bednet type	Insecticide-resistance status	No. of tracks	Average track duration (s)	Average velocity (m/s)
Kisumu	Untreated	Susceptible	7631	22.39 (1.00–698.11)	344.29 (5.20–2290.84)
N’Gousso	Untreated	Susceptible	6125	21.40 (1.00–1010.03)	453.78 (3.54–2602.18)
Banfora	Untreated	Resistant	7189	24.26 (1.00–475.42)	389.57 (0.74–2478.15)
VK7	Untreated	Resistant	5862	17.04 (1.00–457.77)	429.54 (4.10–2102.89)
Kisumu	Olyset	Susceptible	936	18.59 (1.00–317.98)	504.43 (4.33–2709.62)
N’Gousso	Olyset	Susceptible	1801	8.77 (1.00–227.30)	658.58 (5.89–2819.28)
Banfora	Olyset	Resistant	2088	10.59 (1.00–336.33)	589.27 (3.78–2977.75)
VK7	Olyset	Resistant	1986	10.14 (1.00–341.11)	554.50 (1.74–3273.68)

### 2.2 Data processing

The dataset contains trajectories of varying durations, which can bias the ML model by impacting the features generated. This variation in duration can result due to small obstructions in the camera view, the mosquitoes walking or resting on the bednet or mosquitoes leaving the camera view, leading to the trajectories to be broken.

To account for this, we utilised a moving window technique to split tracks into segments and unify track durations (Qureshi et al., 2023). The window size and window overlap of this windowing technique were then optimised for the pipeline through hyperparameter tuning described in *Section* 2.9.

Due to positions being missed, linear interpolation was applied to maintain track continuity. However, in some cases where there were many consecutive interpolated positions or large gaps in tracks, the information quality was reduced. To address this issue, a segment quality metric was used to score each trajectory segment, where a larger score denotes a lower information quality segment (Qureshi et al., 2025). A segment quality threshold was defined to remove segments that are higher than this. To define the threshold, the mutual information of each segment at various quality levels was computed. The weighted average of the thresholds for each segment feature that maximised the information content was used as the final threshold. Segments with quality scores larger than this threshold were removed, resulting in a filtered dataset.

### 2.3 Feature extraction

Features of flight were extracted from the trajectories. Some features describe the kinematic nature of the tracks, capturing the movement dynamics such as the speed, acceleration and turning angles of the mosquito. The other features describe the geometric characteristics and shape of the track, such as the curvature of the track. A statistical summary was computed for each feature for each trajectory segment. The statistical summary only considers real positions (i.e. not interpolated) within a segment. The calculations for the features of flight are detailed in Qureshi et al. (2023, 2025) and provided in Supplementary file 1.

### 2.4 Dataset partitioning

The dataset, containing 40 total experiment trials (23 Olyset trials and 17 untreated trials), was randomly split into two distinct datasets: a modelling set and a tuning set. The tuning set was used for feature selection and hyperparameter tuning, consisting of 19 trials. The modelling set, containing 21 trials, was dedicated to the ML model training and evaluation. Trials were sampled to ensure a balanced representation of all four mosquito strains (Banfora, VK7, Kisumu, and N’Gousso) and both net types (Olyset and untreated) across the two sets. This stratification helped maintain biological and experimental diversity while avoiding overlap. Partitioning the dataset by trials prevents data leakage and better reflects real-world scenarios where entire trials are tested independently. This approach enables robust model training, ensures thorough cross-validation, and maintains the reliability of performance evaluations.

### 2.5 Feature selection

To select meaningful features, the Mann-Whitney *U-*test, a non-parametric statistical test, from SciPy (Virtanen et al., 2020) was used. A family-wise error rate (FWER) controlling procedure using Bonferroni correction was used to reduce group testing *P*-value inflation. Features that rejected the null hypothesis at an FWER < 0.05 were selected. Highly correlated features were removed by calculating the Spearman’s correlation and removing one feature from each pair of highly correlated features (*ρ* > 0.85) from the set of selected features.

### 2.6 Classification model

This study aims to differentiate between behaviours of mosquitoes in the presence of an untreated net (UT) and the Olyset ITN (OL) using a ML classifier. The XGBoost classifier (Chen and Guestrin, 2016), a boosting-tree algorithm, known for being the state-of-the-art in tabular classification, was used. In order to generate whole track predictions, every track segment was classified independently, and the mode of the segment binary predictions for each track was subsequently used as the prediction for the complete track.

Prior to training, each feature was standardised using Z-score normalisation, where the mean and standard deviation values were calculated from the training set. As the training dataset has class imbalance, the *scale_pos_weight* parameter within the XGBoost model was adjusted to be the ratio of the number of UT segments to the number of OL segments.

The decision boundary of the model was also adjusted. Typically, the predictions of a model are considered through the probability score of each class, where in a binary classification task, a probability score above 0.5 for a given class leads to that class being the final prediction. The decision boundary is adjusted by evaluating the training dataset at varying decision boundaries {0.01, 0.02, …, 0.99}. The decision boundary that returns the largest Matthews correlation coefficient score on the training set is used to set the decision boundary threshold on the test.

### 2.7 Model training strategies

The ML model was trained using a subset of trials. Each trial consists of a 2-h-long experiment where mosquito trajectories were generated. For insecticide-susceptible strains on the Olyset net, it was noticed that mosquitoes were killed or highly impacted after 30 min. As such, there were very few tracks generated after 30 min in the experiment, and those that existed were highly distorted and unreliable.

To ensure the ML model was trained on reliable and consistent trajectories, a balanced training strategy (BTS) was employed. This involved training the model using OL IS tracks generated within 30 min of the experiment, as well as sampling OL IR, UT IS, and UT IR tracks so that the number of tracks for each class is approximately equal. Other training strategies were also considered, such as an early training strategy (training the model using tracks generated within 30 min of each experiment) and a comprehensive training strategy (training the model on all available data). Results of these other training strategies can be found in Supplementary file 1, and were similar or slightly worse than the BTS.

### 2.8 Evaluation and interpretation

The ML model was evaluated on the modelling dataset using a trial-based cross-validation approach. In this approach, each fold represents a unique train-test split at the trial level, where the model is trained on a subset of trials and evaluated on a separate, unseen set of trials. Specifically, each fold consisted of 12 training trials and 9 testing trials, selected to ensure a balanced representation of net types and mosquito strains in both sets. Trials were sampled such that each test set included approximately 1 UT trial and 2 OL trials per strain, accounting for the lower number of useable trajectories in OL trials (see Table 1). The remaining trials were used for training. A total of 1944 balanced combinations of 21 modelling trials (comprising 9 UT trials and 12 OL trials) were generated, ensuring even representation across mosquito strains. From these, 30 folds were randomly sampled to evaluate the model’s generalisability. This trial-level cross-validation structure helps prevent data leakage between training and testing sets and reflects real-world scenarios where entire experimental conditions may be encountered for the first time.

Performance metrics were calculated on whole tracks using Scikit-learn (Pedregosa et al., 2011), including balanced accuracy, ROC AUC (area under the receiver operator curve) score, PR AUC (area under the precision-recall curve) score, F1 score, precision score, recall score, Matthew Correlation Coefficient (MCC), Cohen kappa coefficient and log-loss score. The arithmetic mean, minimum and maximum of the performances across all folds was provided.

Confusion matrices provided a visual summary of model performance, showing the normalised percentages of true and false predictions for IR and IS classifications relative to the true labels. High diagonal values indicated correct classifications, while off-diagonal values highlighted errors. Under random chance, each row would display a 50:50 split between classes. Normalising the data made it easier to evaluate performance within each class and identify misclassification patterns.

To explain the model predictions, Shapley Additive exPlanations (SHAP) (Lundberg and Lee, 2017) values are used. These represent the contribution of each feature to predictions made by the ML model. By using this, we can gain insights into the features that have the strongest influence on the model’s output and how they interact with each other. Here, the SHAP values are calculated on track segments, which can aid the understanding of the differences between mosquito behaviours around untreated bednets and the Olyset net.

The impact of insecticide on IS strains acts fairly quickly, thus, it is necessary to explore the changes in accuracy of the ML model across the experiment time. Each trial is 2 h long, so the accuracy for every 5-min period of the experiment can be plotted. For tracks that start within the 5-min segment, the average accuracy was computed and plotted as a line graph. Similarly, performance metrics are calculated for each strain, each class, and before/after 30 min of the experiment.

### 2.9 Hyperparameter tuning

The ML pipeline has various parameters that require optimising to obtain the best performance whilst also reducing overfitting. This includes the window size and the overlap of the windowing technique used to split tracks into segments, as well as the ML model parameters. The parameters were tuned together in a cross-validated grid search approach, attempting to maximise balanced accuracy on an unseen test set. The full set of optimised hyperparameters identified for each model can be found in Supplementary file 1.

## 3 Results

### 3.1 Data processing

Through hyperparameter tuning, a segment size of 7.5 s and a segment overlap of 7 s obtained the best performance on the tuning data set. Using these parameters, the final dataset contains 12,991 tracks, which are split into 881,713 track segments.

### 3.2 Model evaluation

Table 2 provides model performance metrics, including the arithmetic mean, minimum, and maximum values for each metric. Performance indicators include F1 score, recall, precision, and the area under the precision-recall curve (PR AUC). To provide a balanced assessment, metrics were calculated twice, once with the UT class as the positive class, and once with the OL class as the positive class. This approach ensures that evaluation is not biased by class designation and allows symmetrical reporting across both classes. These summary statistics offer a clear view of the model’s variability and stability across different evaluation metrics.

**Table 2:** Performance metrics of the XGBoost classification model on independent test data. Each evaluation metric is reported as the arithmetic mean, with the minimum and maximum values provided in brackets across all cross-validation folds.

Evaluation metric	Performance
Balanced accuracy	0.838 (0.798–0.888)
ROC AUC	0.925 (0.899–0.944)
Matthew Correlation Coefficient	0.502 (0.442–0.574)
Log loss	0.354 (0.288–0.460)
Cohen Kappa Coefficient	0.465 (0.371–0.558)
F1 score (OL)	0.521 (0.427–0.606)
F1 score (UT)	0.935 (0.900–0.953)
Recall (OL)	0.782 (0.683–0.908)
Recall (UT)	0.894 (0.824–0.939)
Precision (OL)	0.397 (0.283–0.538)
Precision (UT)	0.979 (0.967–0.991)
PR AUC (OL)	0.489 (0.395–0.621)
PR AUC (UT)	0.803 (0.771–0.839)

**Fig. 2:**
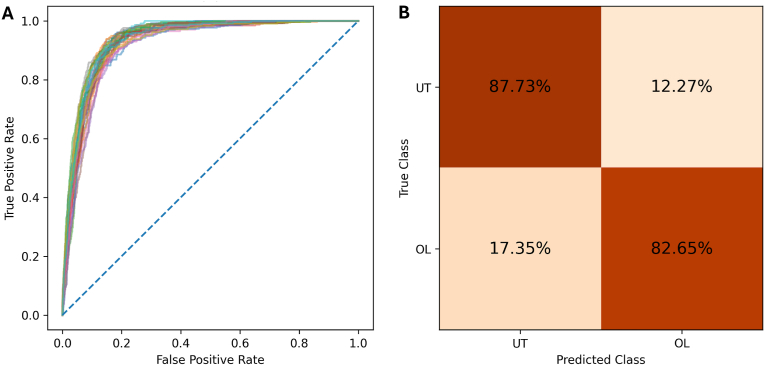
Model performance evaluation classifying mosquito flight trajectories based on net type (untreated *vs* Olyset) across all mosquito strains. **A** Receiver Operating Characteristic (ROC) curve for each fold, illustrating the trade-off between sensitivity (true positive rate) and 1-specificity (false positive rate) across classification thresholds. Each line represents the performance of an individual fold, providing insight into model consistency. **B** Confusion matrix derived from independent test data, displaying the normalised percentages of predictions averaged across all folds. The matrix is normalised by the true class, highlighting the proportion of correct and incorrect predictions within each class. *Abbreviations*: OL, Olyset net; UT, untreated net.

Fig. 2 illustrates the ROC curves with each fold’s ROC curve shown as a different coloured line, as well as the confusion matrix for the balanced method. As the test set was imbalanced, the confusion matrix shows the normalised percentages of the predictions across all folds. The normalisation was performed over the true class such that the proportion of correct predictions can be highlighted.

**Fig. 3:**
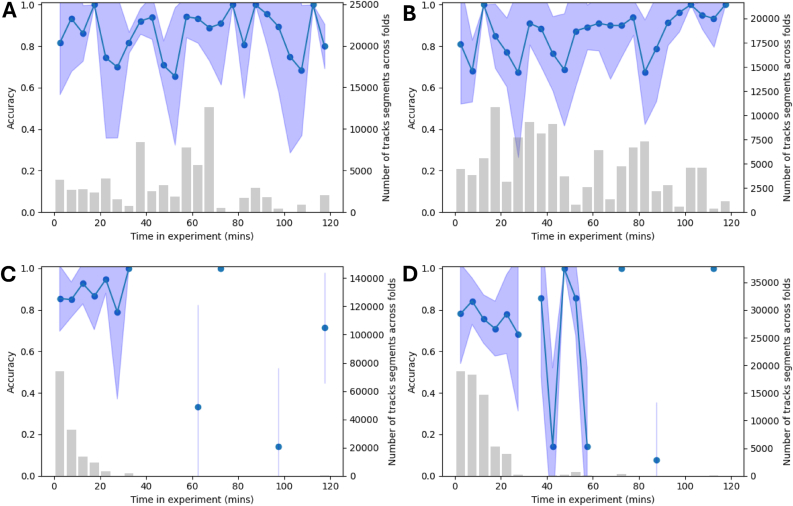
Time-resolved model performance when classifying mosquito flight segments based on net type (Olyset *vs* untreated), shown for each mosquito strain on the Olyset net: Banfora (**A**), VK7 (**B**), Kisumu (**C**), and N’Gousso (**D**). The solid lines and dots represent the average classification accuracy of the model across all cross-validation folds within each 5-min time window of the 2-h experimental period. The shaded blue regions indicate the standard deviation across all folds, providing a measure of variability and robustness. The accompanying bar plots illustrate the total number of track segments analysed across all folds for each strain, highlighting the dataset size and distribution.

**Fig. 4:**
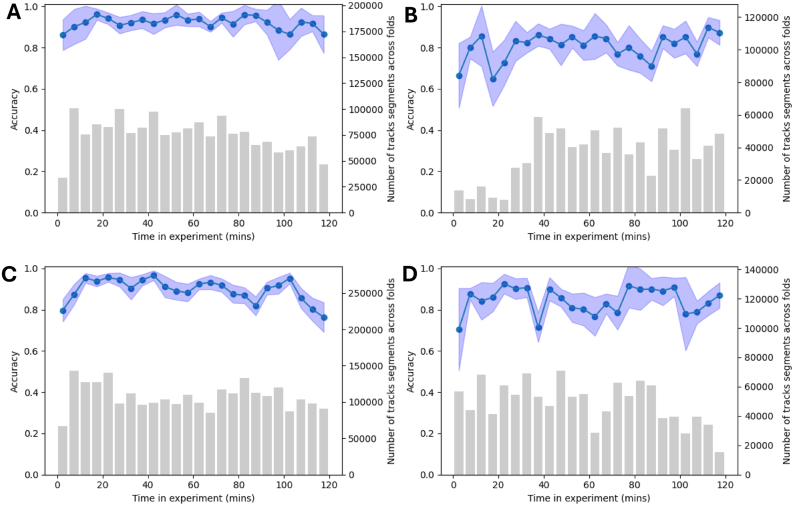
Time-resolved model performance when classifying mosquito flight segments based on net type (Olyset *vs* untreated), shown for each mosquito strain on the untreated net: Banfora (**A**), VK7 (**B**), Kisumu (**C**), and N’Gousso (**D**). The solid lines and dots represent the average classification accuracy of the model across all cross-validation folds within each 5-min time window of the 2-h experimental period. The shaded blue regions indicate the standard deviation across all folds, providing a measure of variability and robustness. The accompanying bar plots illustrate the total number of track segments analysed across all folds for each strain, highlighting the dataset size and distribution.

To examine how classifier performance changes over time, we plotted the average accuracy within each 5-min segment of the 2-h experimental trials for both the OL and UT nets. Fig. 3 shows accuracy trends for the OL net, while Fig. 4 shows trends for the UT net. In these plots, the central line represents the average accuracy across all folds for track segments that start within each 5-min interval. The surrounding blue shaded area represents the standard deviation, highlighting the variation in accuracy across folds. At the Olyset net, larger variability in later time points is primarily due to the decreasing number of available track segments, especially from insecticide-susceptible (IS) mosquitoes, which experience high mortality during the course of the experiment.

**Fig. 5:**
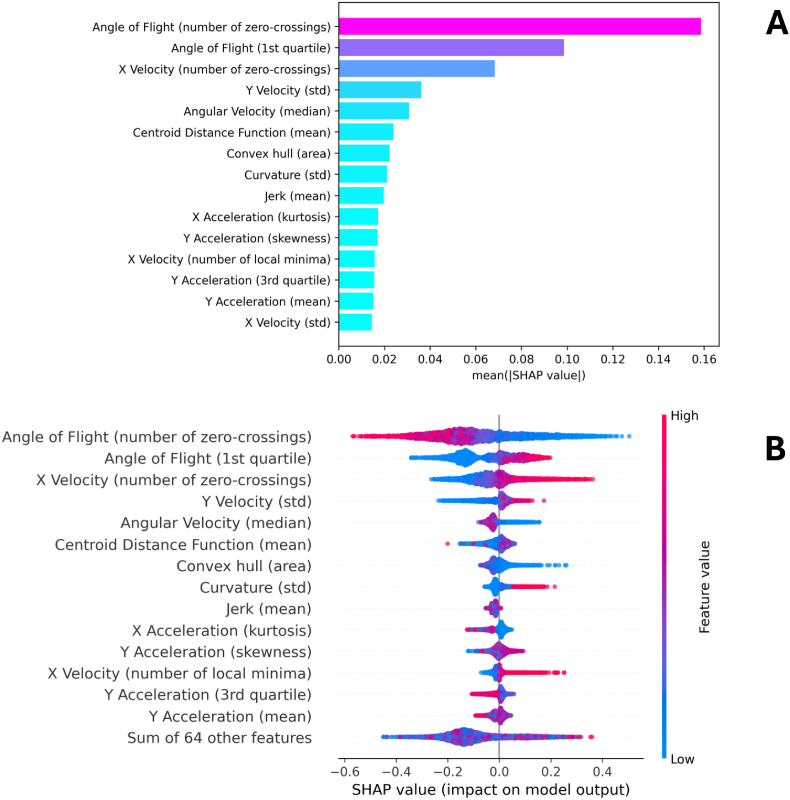
SHAP visualisations for the best-performing model fold applied to independent test data. **A** SHAP bar plot with features ranked based on their mean absolute SHAP values, indicating their overall importance in the model’s predictions. The bar colour corresponds to feature importance using a gradient from the cool-warm colourmap, with higher importance features shown in pink and lower importance features in blue. **B** SHAP summary plot, where each dot represents an individual data segment. The colour gradient of the dots reflects the feature values, with red indicating higher values and blue indicating lower values. In both visualisations, features are sorted by their mean absolute SHAP values. Positive SHAP values indicate contributions towards the Olyset net class, whereas negative SHAP values indicate contributions towards the untreated net class. These plots provide insights into feature influence and directionality within the classification model.

For the best performing fold, SHAP plots are provided in Fig. 5. This includes a SHAP bar plot, and SHAP summary plot. SHAP summary and bar plots for the other training strategies are provided in Supplementary file 1 and display the same trends.

## 4 Discussion

ITNs play a pivotal role in controlling the spread of mosquito-borne diseases like malaria. However, understanding mosquito behaviour in response to insecticides and how these behaviours differ between resistant and susceptible strains can reveal potential areas for improvement in vector control strategies. Our machine learning models displayed an outstanding ability to distinguish mosquito trajectories captured around an untreated net and a treated net, providing insight into the behavioural responses and adaptions made by mosquitoes when around an ITN. Across the three training approaches, model performance remained exceptional with average balanced accuracies between 0.813 and 0.838, and average ROC AUC scores exceeding 0.92. The un-normalised confusion matrices (see Supplementary file 1) appear distorted due to imbalance in the test datasets, as a result of IS mosquitoes at the OL net being killed after 30 min whereas IR mosquitoes are present throughout. It follows that over the 2-h experiments, there is little-to-no variation for UT accuracy, whereas OL accuracy displays more varied performance, shown in Fig. 3, Fig. 4. It was hypothesised that the accuracy on the OL net would be poor initially and then improve with time as the behaviour of the mosquitoes would change from UT to OL. However, the results demonstrate that there is an immediate behavioural response at an OL net for both IR and IS mosquitoes. This could suggest that net contact occurs very quickly as the mosquitoes are attracted to the human bait, or that the OL net has repellent properties (discussed below).

The SHAP analysis identified three dominant features contributing to model predictions: the number of zero-crossings in flight angle change; the first quartile of flight angle change; and the number of zero-crossings in horizontal velocity. Together, these features suggest that mosquitoes interacting with the Olyset net experience highly convoluted flight paths and are likely indicative of disorientation caused by insecticidal exposure.

As the change in angle of flight is always a positive value, the number of zero-crossings in this feature represents the total number of zero values in a track segment. In other words, a high value represents little change in angle within a segment. In the SHAP summary plot (Fig. 5), the trajectory segments with low numbers of zero crossings in angle of flight change tend to contribute towards the OL class, suggesting that tracks on the OL net are more convoluted than UT. Similarly, the higher values of the first quartile of angle of flight change indicate that larger angle changes were experienced within a track segment, and tended towards the OL class. Different to change in angle of flight, horizontal velocity is a signed quantity, and thus the number of zero crossings represents the total number of zero values and zero crossings. A zero crossing in this case means that the mosquito has changed direction in the X direction (across the camera). Larger values of this feature also tend towards the OL net class, where high values suggest more direction changes. These three features account for most of the predictive power of the ML model. Across them all, they suggest that track segments on the OL net are highly convoluted with many large angle changes, as well as more direction changes. Simply put, these tracks are more chaotic as the mosquitoes are impacted by the insecticide.

Previous studies demonstrate conflicting evidence of the repellency of permethrin, the active ingredient in the Olyset net. For example, it was found that permethrin-treated clothing repelled 40–52% of *Aedes albopictus* mosquitoes (Vang et al., 2022), whereas in an experimental hut trial, the results for *An.* *gambiae* at Olyset nets showed the mosquitoes were not repelled (Spitzen et al., 2017). Some of the confusion has arisen from the use of differing definitions for the behavioural reactions of mosquitoes to insecticide (Fatou and Müller, 2024). Here, we define repellency as a pre-contact response, where the presence of the insecticide deters mosquitoes from approaching the treated net. Irritancy, on the other hand, we define as a behavioural response after contact with insecticide-treated surfaces, which may potentially cause disorientation in the mosquito. In the literature, permethrin was found to be repellent in cases where a permethrin solution was created to coat or impregnate a material, either a bednet (Lindsay et al., 1992), test papers (Vang et al., 2022) or textile samples (Yu et al., 2021). In particular, Lindsay et al. (1992) considered the observed repellency to be associated with volatile constituents of the emulsifier concentrate rather than permethrin itself. In contrast, when the permethrin was fused with resin to form the fibres used in the Olyset ITN, a number of studies failed to find evidence for repellency (e.g. Spitzen et al., 2017; Fatou and Müller, 2024). Our model revealed that mosquitoes display sustained disrupted flight behaviour at the OL net compared to a UT net. For individual segments, the classifier exhibited higher noise and it was not possible to reliably examine changes in behaviour before and after a mosquito’s first contact with the Olyset ITN. Therefore, the effective temporal resolution of this ML approach is the trajectory length which has average values between 10.14 and 24.26 s (see Table 1). This period may include first contact with the net and hence a small contribution of repellent behaviours as well as irritancy. Hence, further studies need to be conducted to identify any repellent properties.

Both resistant and susceptible mosquitoes exhibit similarly disrupted flight behaviours around the Olyset net. This is a key finding, as it suggests that resistance may not entirely shield mosquitoes from the behavioural effects of the insecticide, even if it improves their survival. Both IR and IS mosquitoes exhibit convoluted and erratic flight trajectories when exposed to the Olyset net, characterised by frequent direction changes and chaotic movements (see Supplementary file 1: Fig. S10).

While *An. gambiae* (*s.s.*) and *An.* *coluzzii* are morphologically indistinguishable and occupy similar ecological niches, some studies have reported behavioural or physiological distinctions between the two species, such as differences in host-seeking and environmental responsiveness (Akogbéto et al., 2018; Gueye et al., 2023). However, in the context of this study, both net classes (OL and UT) included a balanced representation of both species and resistance statuses, allowing the model to learn behavioural patterns linked to net type independently of species identity. This design reduces the risk that species-level variation confounds classification outcomes. Moreover, recent evidence (Qureshi et al., 2025) has demonstrated that the insecticide-susceptible Kisumu (*An. gambiae* (*s.s.*)) and N’Gousso (*An. coluzzii*) strains exhibit remarkably similar flight behaviours, especially under control (untreated net) conditions. This supports the interpretation that the disrupted flight behaviour observed at the Olyset net is primarily driven by insecticidal exposure, rather than intrinsic species-level differences. The observation that both resistant and susceptible mosquitoes exhibit similarly erratic responses to Olyset further reinforces this conclusion.

Interestingly, within the first 30 min of the experiment, the number of IS trajectory segments at the Olyset net outnumbered those of IR (even though the same number of mosquitoes are released for each trial). The filtered and unfiltered segments exhibited the same trend. The duration and distance travelled by the IS mosquitoes at the OL net were less than those for IR and were shorter than those for IS mosquitoes at an UT net (see Supplementary file 1: Figs. S11-S18). This suggests that IS mosquitoes are disproportionately affected by the insecticide in the early stages, exhibiting more rapid flight paths, possibly in attempts to escape or avoid the net before dying due to insecticide toxicity.

Three distinct training strategies were employed to establish models for mosquito behaviour at the OL and UT nets. The comprehensive strategy included late-stage behaviour when insecticidal effects were at their peak, while the early training strategy excluded these to focus on periods where both IR and IS mosquitoes are active. The balanced training strategy (BTS) attempts to equalise the number of tracks between IR and IS mosquitoes. The focus has been given to the results generated *via* the BTS, which yielded the best performance; nevertheless, the other training strategies returned similar conclusions.

While insecticide resistance continues to challenge the efficacy of traditional vector control tools (Ranson and Lissenden, 2016), our findings indicate that exposure to single-ingredient pyrethroids can alter mosquito flight patterns, even in resistant populations. These observations highlight the potential for leveraging behavioural responses in the design of novel vector control strategies. The ML approach demonstrated here provides a scalable framework for evaluating compounds based on their effects on mosquito behaviour, enabling efficient screening prior to field testing.

While our ML approach revealed novel insights into mosquito behavioural responses to Olyset nets, several limitations warrant consideration in future research. The study focused specifically on *An*. *gambiae* and permethrin-treated nets - future work should be expanded to analyse other important vector species (e.g. the more exophilic sibling species *An*. *arabiensis*) or diurnally active mosquitoes, like the arbovirus vector *Aedes aegypti*, and alternative insecticide classes (or combinations thereof) and the surfaces or materials to which they are applied (e.g. the solid surfaces used for indoor residual praying) to develop a comprehensive understanding of multiple behavioural resistance mechanisms. Regarding laboratory adaptation, we acknowledge that long-term colony maintenance may alter natural behaviours. Although the resistant strains used in this study have been routinely reselected for resistance and genotyped to confirm key mechanisms (e.g. *kdr* mutation and elevated P450 expression) (Williams et al., 2019), they may still differ from wild populations in subtle but important ways. Caution should therefore be exercised when extrapolating our findings to field conditions. Field validation of these behavioural insights remains an important area for future research.

We also note that the model reflects patterns learned from the available data and does not necessarily represent behavioural “truth”. As such, results should be interpreted as the model’s best approximation given the training data, and should be used cautiously as absolute representations of mosquito behaviour. In particular, short-term pre-contact repellency - occurring in the first few seconds of flight prior to any net interaction - was not captured by the current segment-based approach and would be an important area for future investigation. Finally, while our ML model successfully classified flight patterns, the underlying physiological mechanisms driving these behavioural changes remain unclear. Further research is needed to investigate these mechanisms and the factors driving behavioural responses to insecticides.

## 5 Conclusions

In conclusion, this study provides evidence that exposure to Olyset ITNs cause an immediate and noticeable disruption to mosquito flight behaviour, regardless of resistance status. Specific flight features, such as sharp angular changes and frequent direction shifts, were identified as distinguishing mosquitoes interacting with treated *versus* untreated nets, using machine learning and explainable AI techniques. These findings suggest that permethrin, when embedded within Olyset fibres, exerts a primarily irritant rather than repellent effect, inducing chaotic and disorientated trajectories. Importantly, this behavioural disruption occurs rapidly, challenging the assumption that insecticidal impact intensifies only after prolonged exposure. The results presented here highlight the value of trajectory-based behavioural analysis as a scalable and data-driven approach to evaluate vector control interventions. Future work should extend this methodology to other mosquito species, insecticide classes, and net types, and seek to validate these findings under field conditions. This approach has the potential to inform the design and assessment of next-generation vector control tools aimed not only at lethality but also at disrupting host-seeking behaviour.

## CRediT authorship contribution statement

**Yasser M. Qureshi:** Conceptualization, Methodology, Software, Data curation, Visualization, Writing – original draft, Writing – review & editing. **Vitaly Voloshin:** Conceptualization, Methodology, Validation, Writing – review & editing. **Amy Guy:** Validation, Writing – review & editing. **Hilary Ranson:** Validation, Writing – review & editing. **Philip J. McCall:** Validation, Writing – review & editing. **James A. Covington:** Conceptualization, Methodology, Validation, Supervision, Writing – review & editing. **Catherine E. Towers:** Conceptualization, Methodology, Validation, Supervision, Writing – review & editing. **David P. Towers:** Conceptualization, Methodology, Validation, Supervision, Writing – review & editing.

## Ethical approval

This study did not require ethical approval as it involved secondary analysis of anonymised trajectory data previously collected under approved protocols in published studies. No new human or animal subjects were involved in this research.

## Funding

Yasser M. Qureshi was supported by the 10.13039/501100000266EPSRC, grant number EP/T51794X/1.

## Declaration of competing interests

The authors declare that they have no known competing financial interests or personal relationships that could have appeared to influence the work reported in this paper.

## Data Availability

Data are available on request from LSTM *via* Professor Philip McCall (Philip.McCall@lstmed.ac.uk). Code is publicly available on GitHub: https://github.com/yasserqureshi1/ut-vs-ol.

## References

[bib1] Akogbéto M.C., Salako A.S., Dagnon F., Aïkpon R., Kouletio M., Sovi A. (2018). Blood-feeding behaviour comparison and contribution of *Anopheles coluzzii* and *Anopheles gambiae*, two sibling species living in sympatry, to malaria transmission in Alibori and Donga region, northern Benin, West Africa. Malar. J..

[bib2] Bhatt S., Weiss D.J., Cameron E., Bisanzio D., Mappin B., Dalrymple U. (2015). The effect of malaria control on *Plasmodium falciparum* in Africa between 2000 and 2015. Nature.

[bib3] Carnevale P., Gay F. (2019). Insecticide-treated mosquito nets. Methods Mol. Biol..

[bib4] Chen T., Guestrin C. (2016). Proceedings of the 22nd ACM SIGKDD International Conference on Knowledge Discovery and Data Mining, San Francisco, California, USA.

[bib5] Chilakam N., Lakshminarayanan V., Keremutt S., Rajendran A., Thunga G., Poojari P.G. (2023). Economic burden of mosquito-borne diseases in low- and middle-income countries: Protocol for a systematic review. JMIR Res. Protoc..

[bib6] Fatou M., Müller P. (2024). 3D video tracking analysis reveals that mosquitoes pass more likely through holes in permethrin-treated than in untreated nets. Sci. Rep..

[bib7] Gleave K., Guy A., Mechan F., Emery M., Murphy A., Voloshin V. (2023). Impacts of dual active-ingredient bed nets on the behavioural responses of pyrethroid resistant *Anopheles gambiae* determined by room-scale infrared video tracking. Malar. J..

[bib8] Gueye O.K., Niang A., Faye M.B., Dia A.K., Ahmed A.A., Sy O. (2023). Characterization of the swarming behavior of *Anopheles coluzzii* and *Anopheles gambiae* (Diptera: Culicidae) populations in a hybrid zone of Senegal. J. Med. Entomol..

[bib9] Hall M.L., Gleave K., Hughes A., McCall P.J., Towers C.E., Towers D.P. (2022). The application of digital holography for accurate three-dimensional localisation of mosquito-bednet interaction. Light Adv. Manufact..

[bib10] Ito T., Okuno T. (2006). Development of ‘Olyset® Net’ as a tool for malaria control.

[bib11] Jones J., Murray G.P.D., McCall P.J. (2021). A minimal 3D model of mosquito flight behaviour around the human baited bed net. Malar. J..

[bib12] Lindsay S.W., Adiamah J.H., Armstrong J.R.M. (1992). The effect of permethrin-impregnated bednets on house entry by mosquitoes (Diptera: Culicidae) in the Gambia. Bull. Entomol. Res..

[bib13] Liu N. (2015). Insecticide resistance in mosquitoes: Impact, mechanisms, and research directions. Annu. Rev. Entomol..

[bib14] Lundberg S.M., Lee S.-I., Guyon I., Luxburg U.V., Bengio S., Wallach H., Fergus R., Vishwanathan S. (2017). Advances in Neural Information Processing Systems.

[bib15] Ngongang-Yipmo E.S., Tchouakui M., Menze B.D., Mugenzi L.M.J., Njiokou F., Wondji C.S. (2022). Reduced performance of community bednets against pyrethroid-resistant *Anopheles funestus* and *Anopheles gambiae*, major malaria vectors in Cameroon. Parasites Vectors.

[bib16] Parker J.E.A., Angarita-Jaimes N., Abe M., Towers C.E., Towers D., McCall P.J. (2015). Infrared video tracking of *Anopheles gambiae* at insecticide-treated bed nets reveals rapid decisive impact after brief localised net contact. Sci. Rep..

[bib17] Pedregosa F., Varoquaux G., Gramfort A., Michel V., Thirion B., Grisel O. (2011). Scikit-learn: Machine learning in Python. J. Machine Learning Res..

[bib18] Qureshi A.I. (2018). Zika Virus Disease.

[bib19] Qureshi Y.M., Voloshin V., Facchinelli L., McCall P.J., Chervova O., Towers C.E. (2023). Finding a husband: Using explainable AI to define male mosquito flight differences. Biology (Basel).

[bib20] Qureshi Y.M., Voloshin V., Gleave K., Ranson H., McCall P.J., Covington J.A. (2025). Discrimination of inherent characteristics of susceptible and resistant strains of *Anopheles gambiae* by explainable artificial intelligence analysis of flight trajectories. Sci. Rep..

[bib21] Qureshi Y.M., Voloshin V., Towers C.E., Covington J.A., Towers D.P. (2024). Double vision: 2D and 3D mosquito trajectories can be as valuable for behaviour analysis *via* machine learning. Parasites Vectors.

[bib22] Ranson H., Lissenden N. (2016). Insecticide resistance in African *Anopheles* mosquitoes: A worsening situation that needs urgent action to maintain malaria control. Trends Parasitol..

[bib23] Spitzen J., Koelewijn T., Mukabana W.R., Takken W. (2017). Effect of insecticide-treated bed nets on house-entry by malaria mosquitoes: The flight response recorded in a semi-field study in Kenya. Acta Trop..

[bib24] Vang A., White A.V., Balanay J.A.G., Tutor Marcom R., Richards S.L. (2022). Evaluation of surface versus total permethrin content in permethrin-treated clothing: Implications for protection against mosquitoes. Pathog. Glob. Health.

[bib25] Virtanen P., Gommers R., Oliphant T.E., Haberland M., Reddy T., Cournapeau D. (2020). SciPy 1.0: Fundamental algorithms for scientific computing in Python. Nat. Methods.

[bib26] Voloshin V., Kröner C., Seniya C., Murray G.P.D., Guy A., Towers C.E. (2020). Diffuse retro-reflective imaging for improved video tracking of mosquitoes at human baited bednets. R. Soc. Open Sci..

[bib27] Williams J., Cowlishaw R., Sanou A., Ranson H., Grigoraki L. (2022). *In vivo* functional validation of the V402L voltage gated sodium channel mutation in the malaria vector *An. gambiae*. Pest Manag. Sci..

[bib28] Williams J., Flood L., Praulins G., Ingham V.A., Morgan J., Lees R.S. (2019). Characterisation of *Anopheles* strains used for laboratory screening of new vector control products. Parasites Vectors.

[bib29] WHO (2016). World Health Organisation Technical Report Series.

[bib30] Yu J.J., Bong L.J., Panthawong A., Chareonviriyaphap T., Neoh K.B. (2021). Repellency and contact irritancy responses of *Aedes aegypti* (Diptera: Culicidae) against deltamethrin and permethrin: A cross-regional comparison. J. Med. Entomol..

