# Supplementary material for: Machine learning reveals immediate disruption in mosquito flight when exposed to Olyset nets
Source: Curr Res Parasitol Vector Borne Dis. 2025 May 22;7:100273. doi: 10.1016/j.crpvbd.2025.100273 (PMC12174569; doi:10.1016/j.crpvbd.2025.100273)
Supplement: Multimedia component 1 [file mmc1.pdf]

Supplementary Information

Machine learning reveals immediate disruption in mosquito flight when exposed to Olyset nets

Yasser M. Qureshi[a*], Vitaly Voloshin[a, b], Amy Guy[c], Hilary Ranson[c], Philip J. McCall[c], James A. Covington[a], Catherine E. Towers[a], David P. Towers[a]

[a] *School of Engineering, University of Warwick, Coventry, CV4 7AL, UK*

[b] *School of Biological and Behavioural Sciences, Queen Mary University of London, E1 4NS, UK*

[c] *Vector Biology Department, Liverpool School of Tropical Medicine, Pembroke Place, Liverpool, L3 5QA, UK*

*Corresponding author:

E-mail address: Yasser.Qureshi@warwick.ac.uk (YMQ)

Feature Calculations

1. Velocity, acceleration and Jerk

These are calculated using second-order central finite difference methods. The signed values are used – where in the case of velocity, a positive velocity indicates motion from head-to-toe of the human bait within the experiment. The axial values are also utilised.

2. Angle of Flight

The angle of flight, α_i, can be calculated using the dot product of two vectors. It describes the relative change in direction between positions.

$$v_i = \begin{bmatrix} x_i - x_{i-1} \\ y_i - y_{i-1} \end{bmatrix}$$

$$v_{i+1} = \begin{bmatrix} x_{i+1} - x_i \\ y_{i+1} - y_i \end{bmatrix}$$

$$\alpha_i = \arccos\left(\frac{v_i \cdot v_{i+1}}{\sqrt{(v_i \cdot v_i)(v_{i+1} \cdot v_{i+1})}}\right)$$

3. Angular Velocity and Angular Acceleration

The angular velocity and angular acceleration are both calculated using second-order finite difference methods. The arctangent function is used to calculate the angle between positions, then the change in angle between consecutive positions is computed. Angular velocity is calculated by dividing this change in angle by the corresponding time difference.

4. Orthogonal Components of Velocity

This is a group of features that describe the tendency of a movement to head in a given direction. The persistence velocity describes the tendency to head tangential to the trajectory while the turning velocity describes the tendency to head normal to the trajectory. It involves converting from the Cartesian to the circular coordinate system:

$$\rho_i = \sqrt{(x_{i+1} - x_i)^2 + (y_{i+1} - y_i)^2}$$

$$\theta_i = \arctan\left(\frac{y_{i+1} - y_i}{x_{i+1} - x_i}\right)$$

The change in θ and instantaneous velocity is calculated:

$$\Theta_i = |\theta_{i+1} - \theta_i|$$

$$v_i = \frac{\rho_i}{t_{i+1} - t_i}$$

Then converting back to the Cartesian coordinate system:

$$P_i = v_i \cos\Theta$$

$$T_i = v_i \sin\Theta$$

Where P_i and T_i are the persistence and turning velocities, respectively.

5. Tortuosity

This is the ratio of the actual distance travelled and the shortest distance between the start and end positions.

$$S = \frac{\sum_{i=0}^{N} \sqrt{(x_{i+1} - x_i)^2 + (y_{i+1} - y_i)^2}}{\sqrt{(x_N - x_0)^2 + (y_N - y_0)^2}}$$

6. Convex Hull

The convex hull is the set of points that forms the smallest possible convex polygon that bounds all the points of a track. From the polygon that is formed, the area and perimeter is extracted and used as features.

7. Centroid Distance Function

This calculated the distances of each position in a track to the centre point of the track.

$$x_c = \frac{1}{N}\sum_{i=0}^{N} x_i$$

$$y_c = \frac{1}{N}\sum_{i=0}^{N} y_i$$

$$C_i = \sqrt{(x_i - x_c)^2 + (y_i - y_c)^2}$$

8. Curvature

Curvature is a measure that defines the deviation of a trajectory from a straight line. It is calculated as:

$$k_i = \frac{\dot{x}_i\ddot{y}_i - \dot{y}_i\ddot{x}_i}{\left(\dot{x}_i^{\,2} + \dot{y}_i^{\,2}\right)^{\frac{3}{2}}}$$

9. Curvature Scale Space

This measures the inflection points in a trajectory at difference scales and is robust to affine transformations. To compute the curvature at varying levels, the trajectory is convolved with a 1D gaussian kernel of width σ. The value of σ is varies, and the zero-crossings locations in the curvature are recorded. The locations of zero-crossings at different σ levels can be plotted as a CSS image. A 1D signal is generated by taking column maximums from this CSS image.

10. Fractal Dimension

Fractal dimension is a measure of the complexity of a trajectory. Values around 1 describe a linear path with a shape similar to a straight line, and values around 2 indicate convoluted movement with a shape similar to a plane. To compute fractal dimension, d, the following equations are used:

$$d = \frac{\log n}{\log \frac{1}{S}}$$

Where n is the number of minature pieces in the path, S is the scaling factor, and d is the fractal dimension. For a trajectory, the values of n and S are defined as follows:

$$n = \sum_{i=0}^{N} \sqrt{(x_{i+1} - x_i)^2 + (y_{i+1} - y_i)^2}$$

$$S = \frac{1}{\sqrt{(\max x - \min x)^2 + (\max y - \min y)^2}}$$

Penalty functions and thresholds assessed.

To remove tracks with substantial gaps (many missing positions), a segment quality metric was used to score track segments. A mutual information-based method was used to obtain a threshold for the segment quality metric.

This is where we developed a penalty function to score a track on its information content. Let $segment$ be a list of length N where each element in the list is either 1 or 0, indicating a real position (1) or an interpolated position (0) for a segment. The penalty function $P(segment, n, m)$ is defined as:

$$P(segment, n, m) = \frac{1}{N}\sum_{i=0}^{N}\begin{cases} n \cdot m^{c_i}, & \text{if } x_i = 0 \\ 0, & \text{if } x_i = 1 \end{cases}$$

Where c_i is the length of the consecutive artificial positions starting from position i until a real position is encountered. If $x_i = 1$, c_i is reset to 0. Various values of n and m were assessed with $n = 1$ and $m = 1.05$ being used for the final model.

To obtain a threshold for the penalty score, a threshold was determined by computing the mutual information at various score thresholds. Then the weighted average threshold is obtained based on the maximum mutual information and its corresponding threshold for each feature. This weighted average is the final score threshold.

Supplementary Table S1. Hypertuning parameter ranges

Parameter	Parameter values
Window size	0.5, 1, 1.5, 2, 2.5, 3, 3.5, 4, 4.5, 5, 5.5, 6, 6.5, 7, 7.5, 8, 8.5, 9, 9.5
Window overlap	0.5, 1, 1.5, 2, 2.5, 3, 3.5, 4, 4.5, 5, 5.5, 6, 6.5, 7, 7.5, 8, 8.5, 9
Learning rate	0.01, 0.1, 0.3
N estimators	50, 100, 150, 200, 250
Max depth	3, 5, 7
Subsample	0.5, 0.7, 0.9
Colsample bytree	0.5, 0.7, 0.9
Reg alpha	0, 0.01, 0.1
Reg lambda	0, 0.01, 0.1
Min child weight	1, 5, 15

Supplementary Table S2. Final hyperparameters for each training strategy.

Parameter	Comprehensive	Balanced	Early
Window size	7	7.5	8
Window overlap	6.5	7	7
Learning rate	0.3	0.3	0.3
N estimators	200	100	200
Max depth	3	5	3
Subsample	0.8	0.7	0.8
Colsample bytree	0.9	0.8	0.9
Reg alpha	0	0.1	0.1
Reg lambda	0	0.1	0
Min child weight	10	20	10

Supplementary Table S3. Performance metrics for the comprehensive and early data training strategies.

Performance metric	Comprehensive data training	Early data training
Balanced accuracy	0.813 (0.769–0.863)	0.838 (0.785–0.885)
ROC AUC	0.931 (0.903–0.952)	0.925 (0.897–0.945)
Matthew Correlation Coefficient	0.521 (0.458–0.618)	0.498 (0.432–0.562)
Log loss	0.323 (0.287–0.361)	0.360 (0.290–0.453)
Cohen Kappa Coefficient	0.506 (0.441–0.615)	0.459 (0.356–0.556)
F1 score (OL)	0.554 (0.490–0.654)	0.516 (0.414–0.603)
F1 score (UT)	0.950 (0.938–0.960)	0.933 (0.902–0.953)
Recall (OL)	0.696 (0.595–0.809)	0.785 (0.656–0.904)
Recall (UT)	0.929 (0.897–0.962)	0.892 (0.831–0.938)
Precision (OL)	0.466 (0.355–0.625)	0.391 (0.273–0.534)
Precision (UT)	0.972 (0.958–0.987)	0.979 (0.964–0.991)
PR AUC (OL)	0.526 (0.392–0.659)	0.489 (0.392–0.612)
PR AUC (UT)	0.799 (0.763–0.833)	0.803 (0.772–0.839)

Supplementary Table S4. Windowing parameters for each model alongside the final number of tracks and segments.

Training Strategy	Window Size (s)	Window Overlap (s)	Final number of tracks	Number of segments
Comprehensive data training	7	6.5	13,469	1,067,889
Early data training	8	7	12,584	440,118
Balanced data training	7.5	7	12,991	881,713

Supplementary Table S5. Accuracy of the XGBoost classification model when applied to independent test data with differing data training strategies for resistant and susceptible strains, before and after 30 minutes.

Training strategy	Comprehensive	Early	Balanced
OL IR (<30min)	0.921 (0.803–1.000)	0.912 (0.750–1.000)	0.929 (0.812–1.000)
OL IS (<30min)	0.836 (0.647–0.958)	0.844 (0.669–0.934)	0.877 (0.727–0.961)
OL IR (>30min)	0.894 (0.791–0.942)	0.889 (0.805–0.967)	0.905 (0.827–0.962)
OL IS (>30min)	0.705 (0.000–1.000)	0.667 (0.000–1.000)	0.752 (0.000–1.000)
UT IR (<30min)	0.859 (0.816–0.904)	0.866 (0.797–0.927)	0.852 (0.802–0.905)
UT IS (<30min)	0.893 (0.860–0.933)	0.899 (0.865–0.936)	0.883 (0.843–0.915)
UT IR (>30mins)	0.854 (0.821–0.879)	0.849 (0.805–0.878)	0.844 (0.800–0.877)
UT IR (>30min)	0.852 (0.814–0.873)	0.834 (0.792–0.871)	0.836 (0.802–0.857)

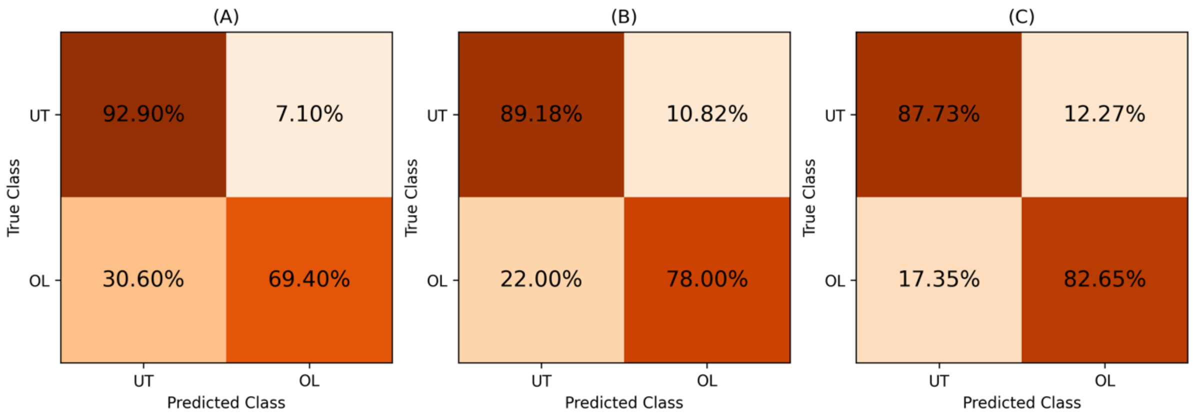

Supplementary Figure S1. Confusion matrices from independent test data displaying the normalised percentages of the predictions of the true class over all folds. In the figure, (**A**) displays the comprehensive data training strategy, (**B**) is the early data training strategy, and (**C**) is the balanced data training strategy.

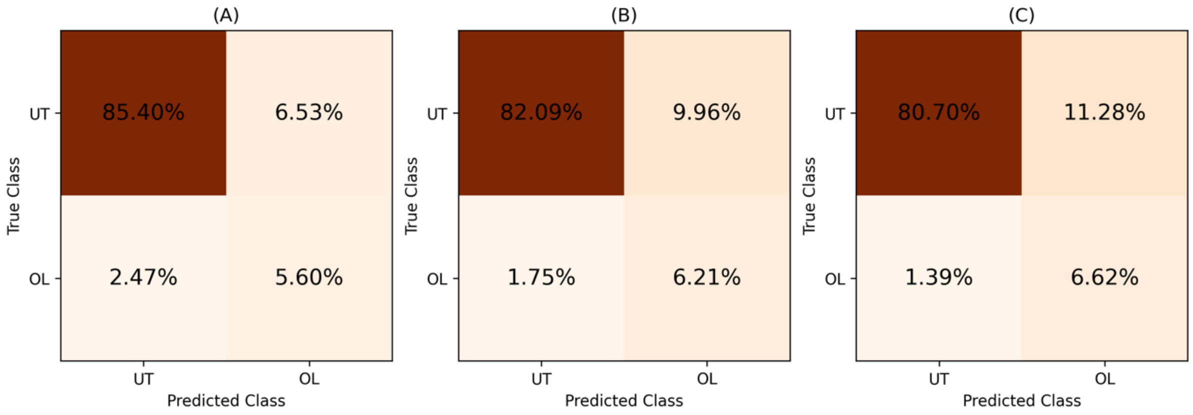

Supplementary Figure S2. Confusion matrices from independent test data displaying the percentages of the predictions over all folds. In the figure, (**A**) displays the comprehensive data training strategy, (**B**) is the early data training strategy, and (**C**) is the balanced data training strategy.

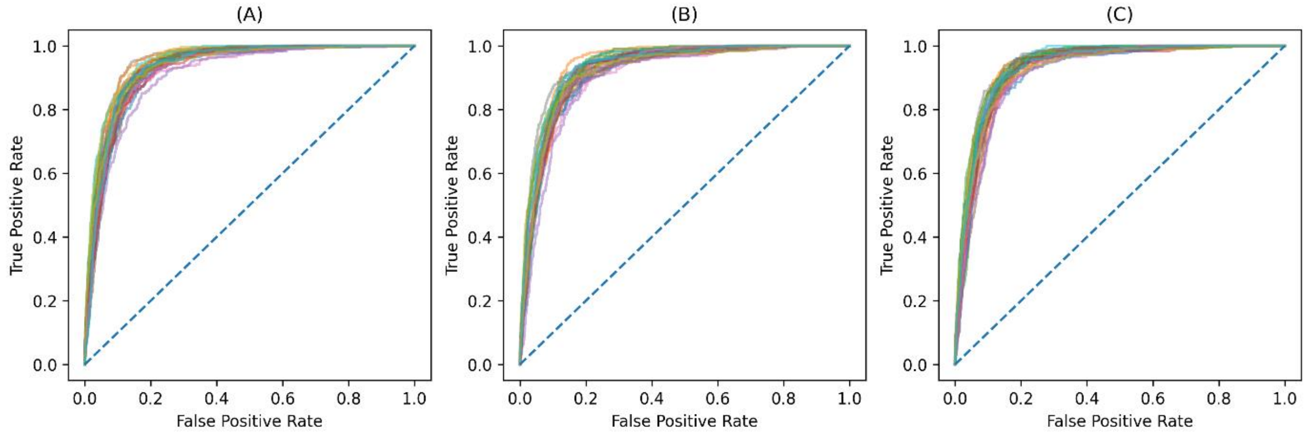

Supplementary Figure S3. ROC Curves from independent test data displaying different coloured lines for each folds' ROC curve. In the figure, (**A**) displays the comprehensive data training strategy, (**B**) is the early data training strategy, and (**C**) is the balanced data training strategy.

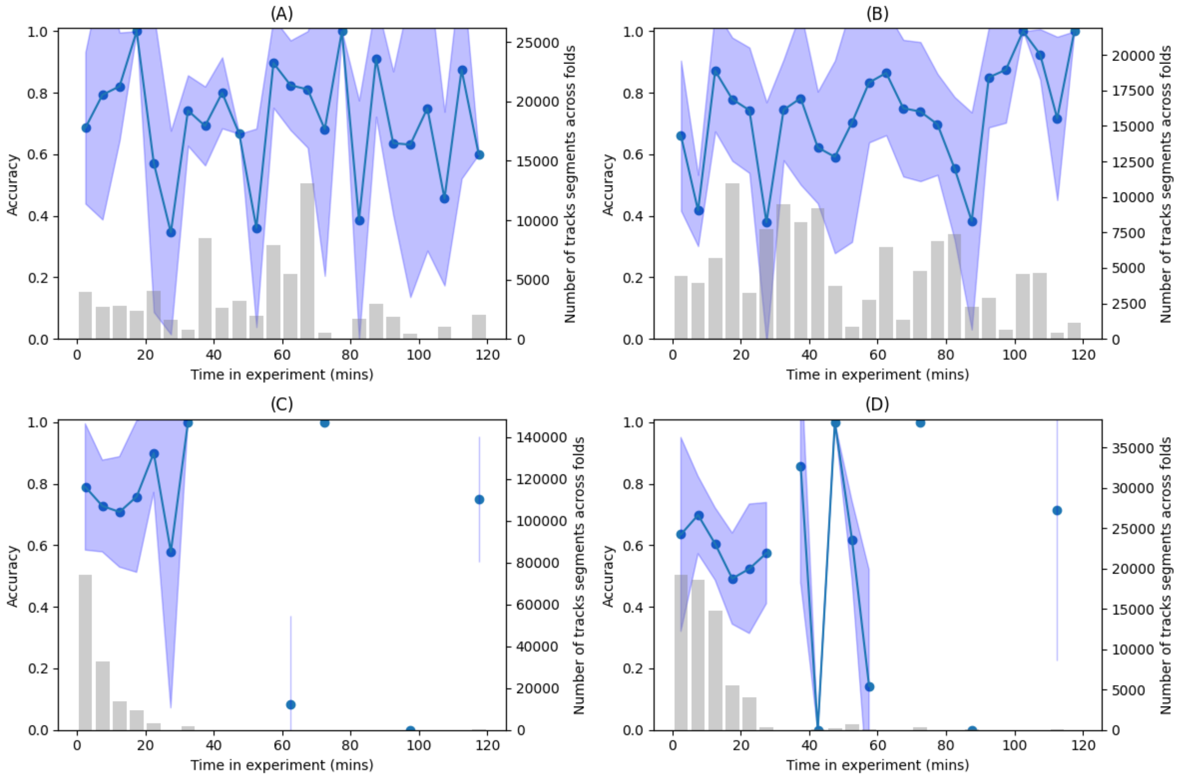

Supplementary Figure S4. Model performance across the experiments for the comprehensive data training strategy for each strain on the Olyset net – (**A**) Banfora, (**B**) VK7, (**C**) Kisumu, and (**D**) Ngoussu. The lines and dots indicate the model accuracy in classification, whilst the bar plot displays the sum of the number of tracks across all folds.

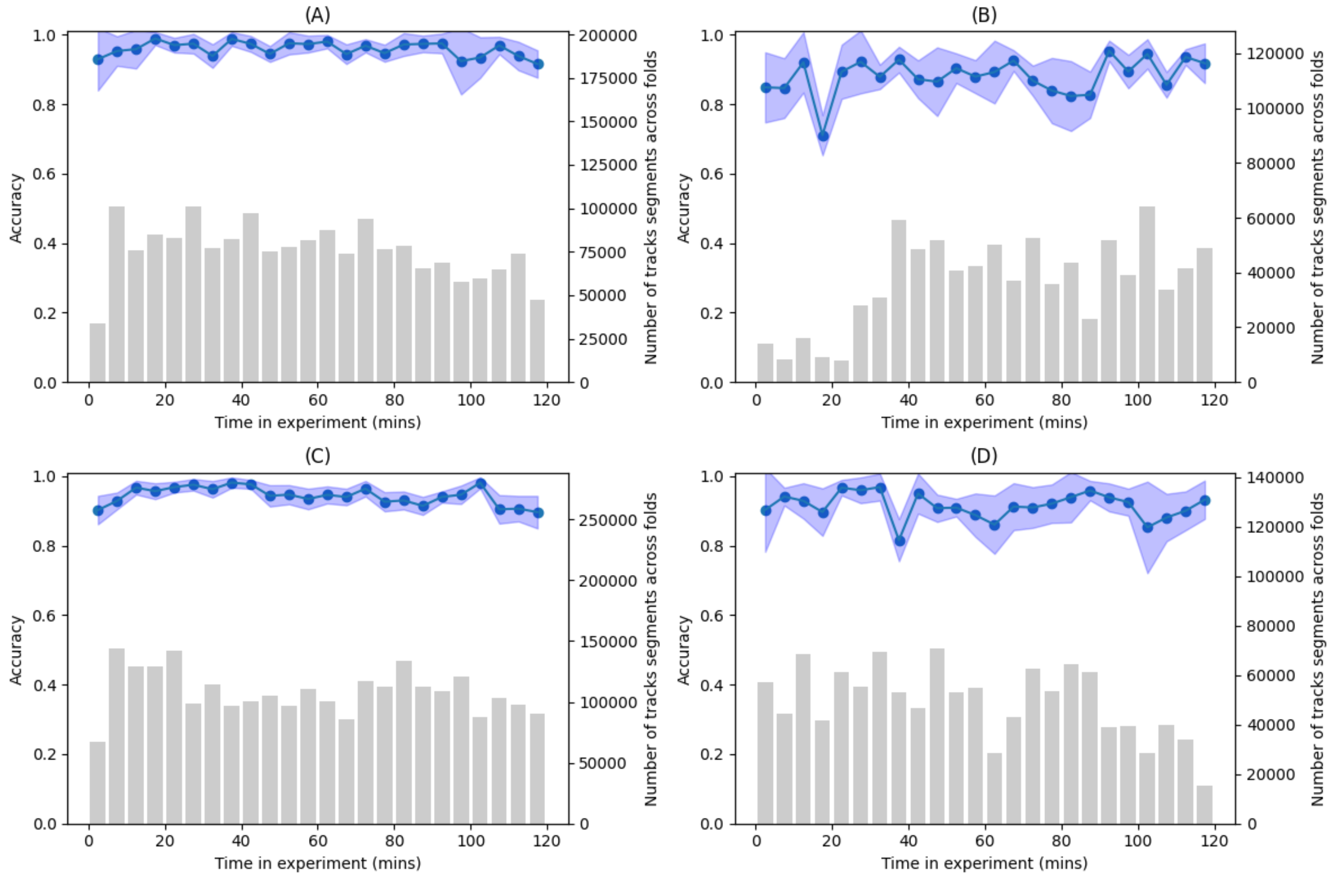

Supplementary Figure S5. Model performance across the experiments for the comprehensive data training strategy for each strain on the untreated net – (**A**) Banfora, (**B**) VK7, (**C**) Kisumu, and (**D**) Ngoussu. The lines and dots indicate the model accuracy in classification, whilst the bar plot displays the sum of the number of tracks across all folds.

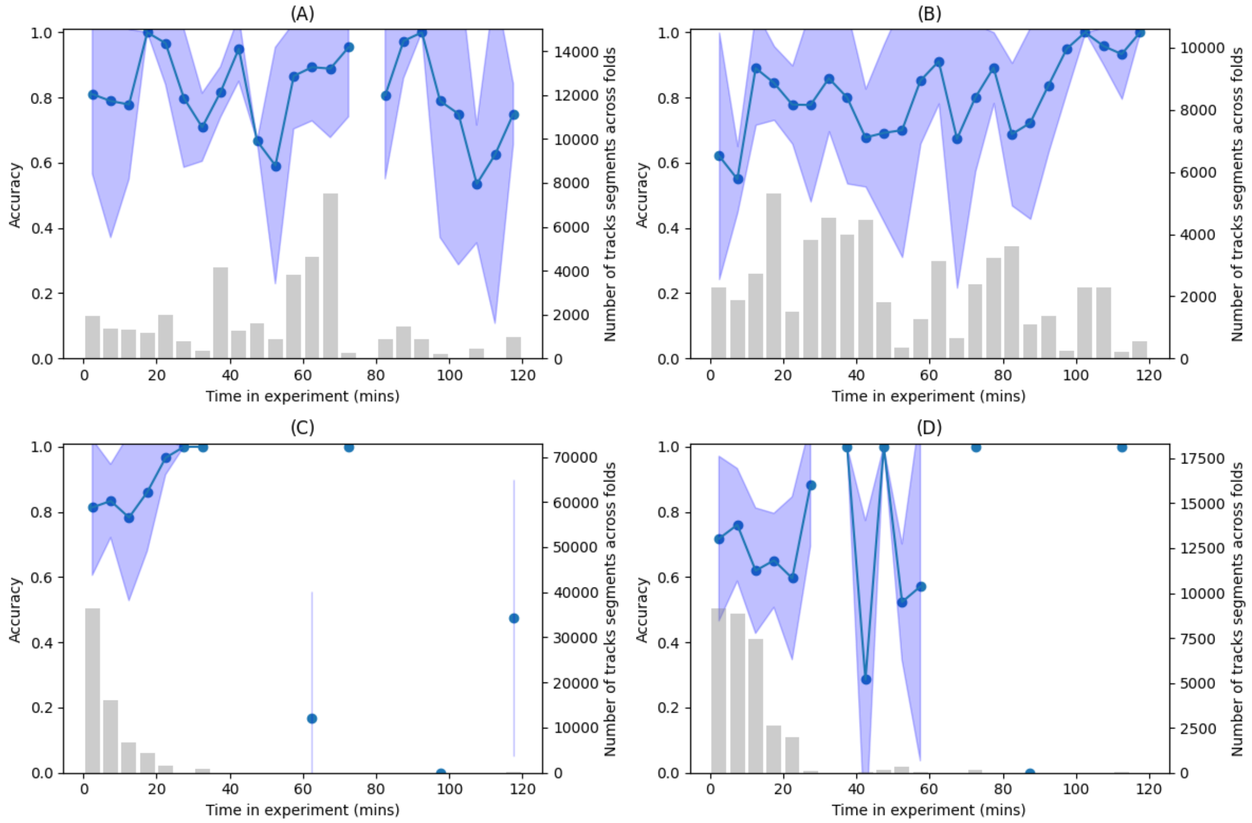

Supplementary Figure S6. Model performance across the experiments for the early data training strategy for each strain on the Olyset net – (**A**) Banfora, (**B**) VK7, (**C**) Kisumu, and (**D**) Ngoussu. The lines and dots indicate the model accuracy in classification, whilst the bar plot displays the sum of the number of tracks across all folds.

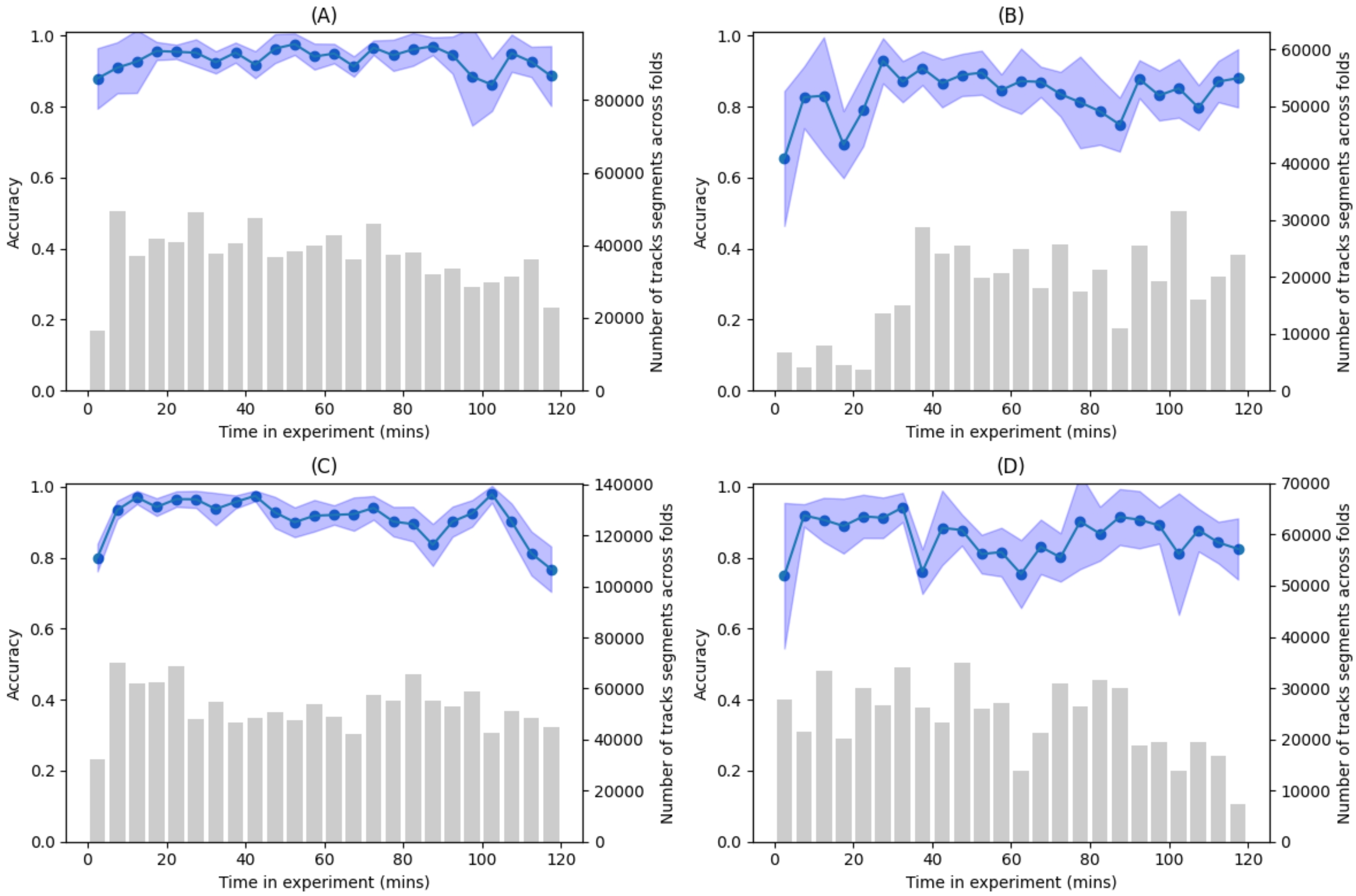

Supplementary Figure S7. Model performance across the experiments for the early data training strategy for each strain on the Untreated net – (**A**) Banfora, (**B**) VK7, (**C**) Kisumu, and (**D**) Ngoussu. The lines and dots indicate the model accuracy in classification, whilst the bar plot displays the sum of the number of tracks across all folds.

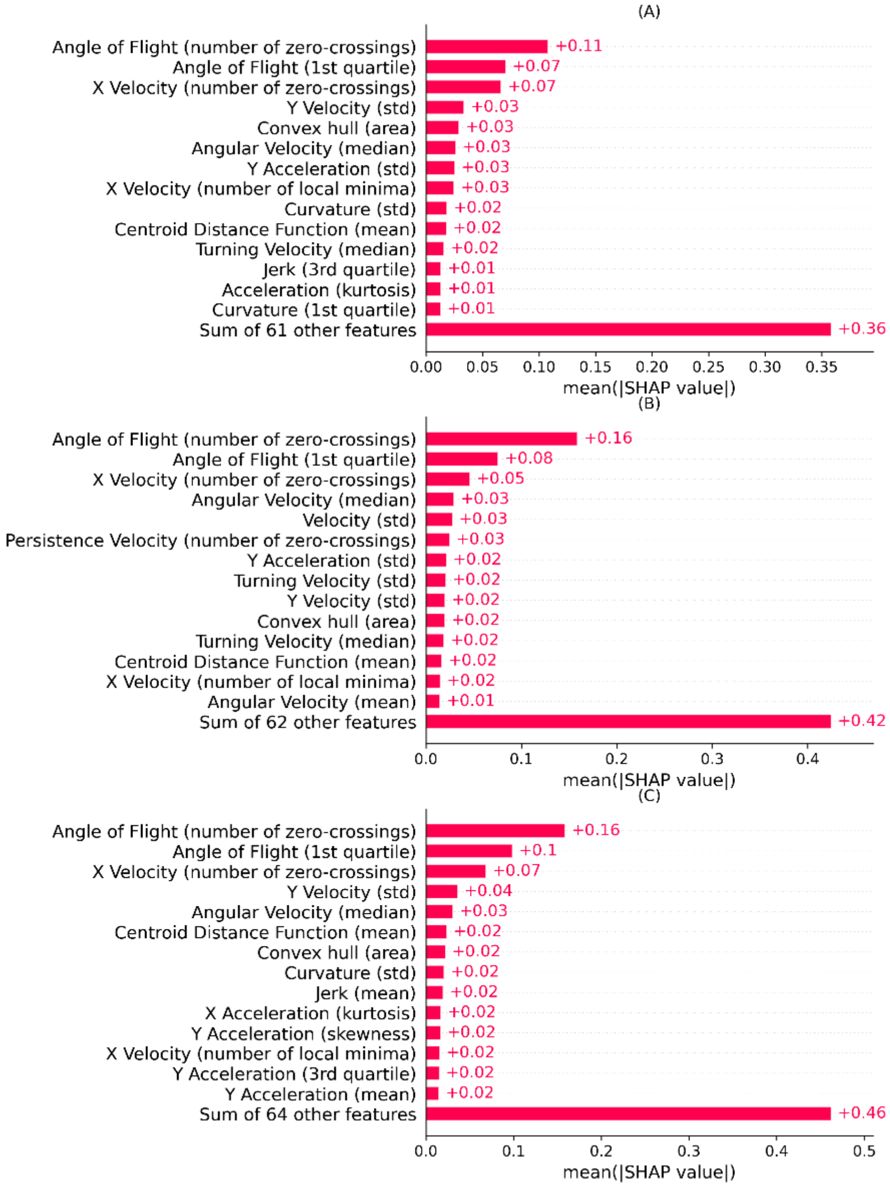

Supplementary Figure S8. SHAP bar plots for the best model fold applied to independent test data for each data training strategy. In the figure, (**A**) displays the comprehensive data training strategy, (**B**) is the early data training strategy, and (**C**) is the balanced data training strategy. The features are ordered by mean absolute SHAP value.

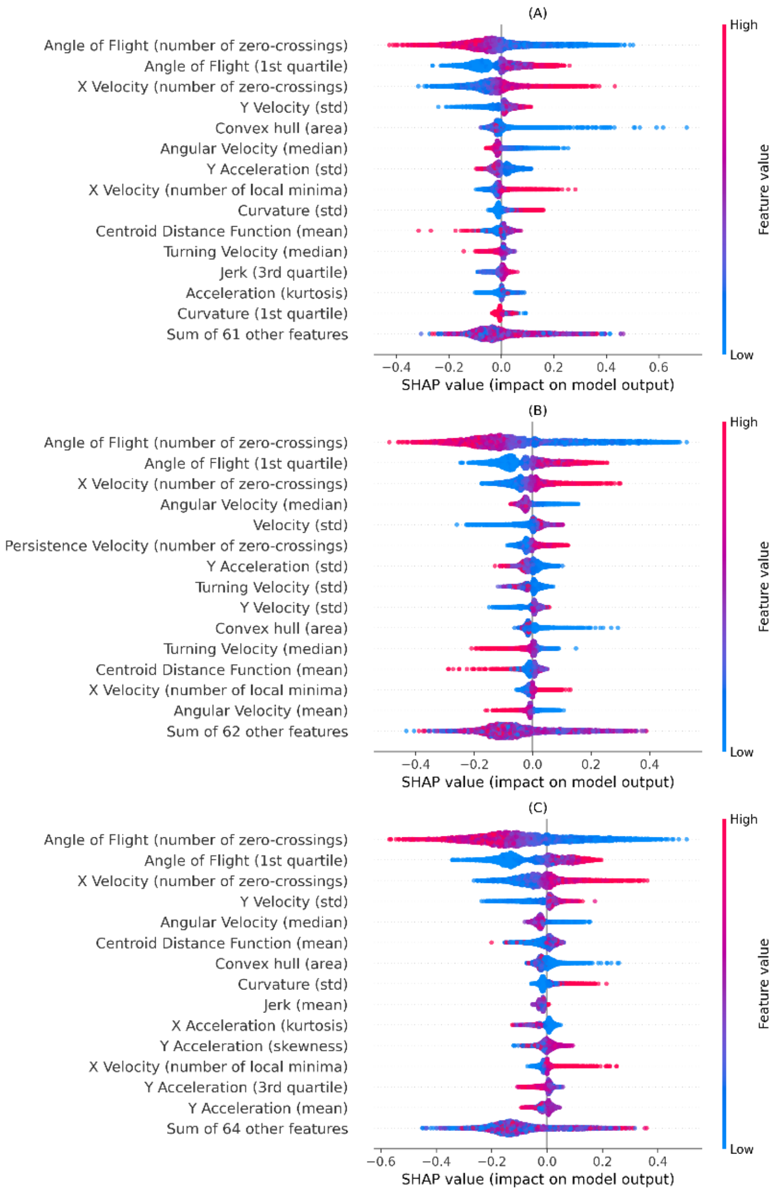

Supplementary Figure S9. SHAP summary plots for the best model fold applied to independent test data for each data training strategy. Features are ordered by mean absolute SHAP value where each dot represents a segment, with its colour displaying its feature value. Positive SHAP values contribute towards the OL class, whilst negative SHAP values contribute towards the UT class. In the figure, (**A**) displays the comprehensive data training strategy, (**B**) is the early data training strategy, and (**C**) is the balanced data training strategy.

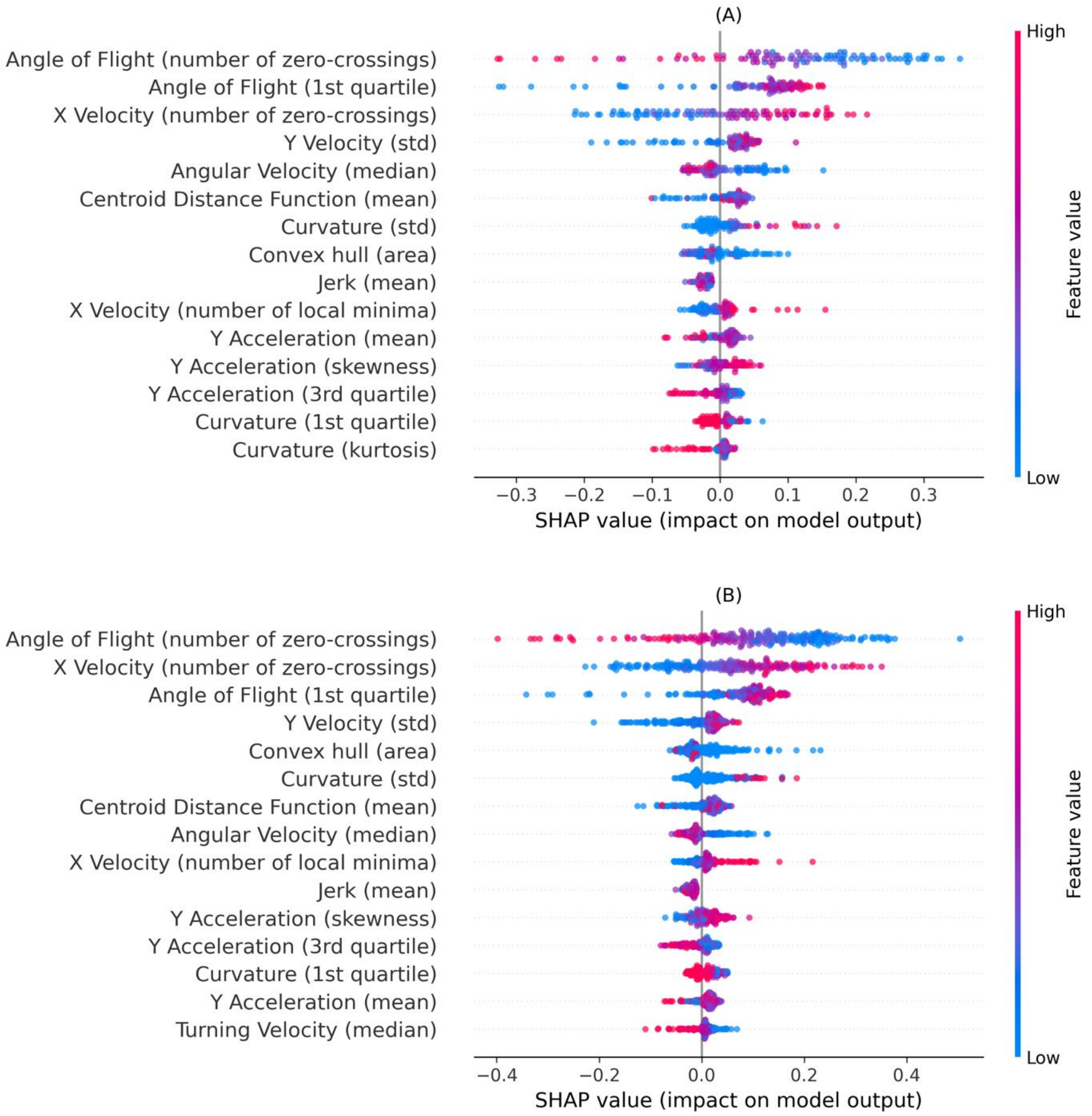

Supplementary Figure S10. SHAP summary plots with IR and IS trajectories shown separately for the best model fold applied to independent test data using the balanced training strategy on the OL net. Features are ordered by mean absolute SHAP value where each dot represents a segment, with its colour displaying its feature value. Positive SHAP values contribute towards the OL class, whilst negative SHAP values contribute towards the UT class. In the figure, (**A**) displays the IS class, and (**B**) displays the IR class.

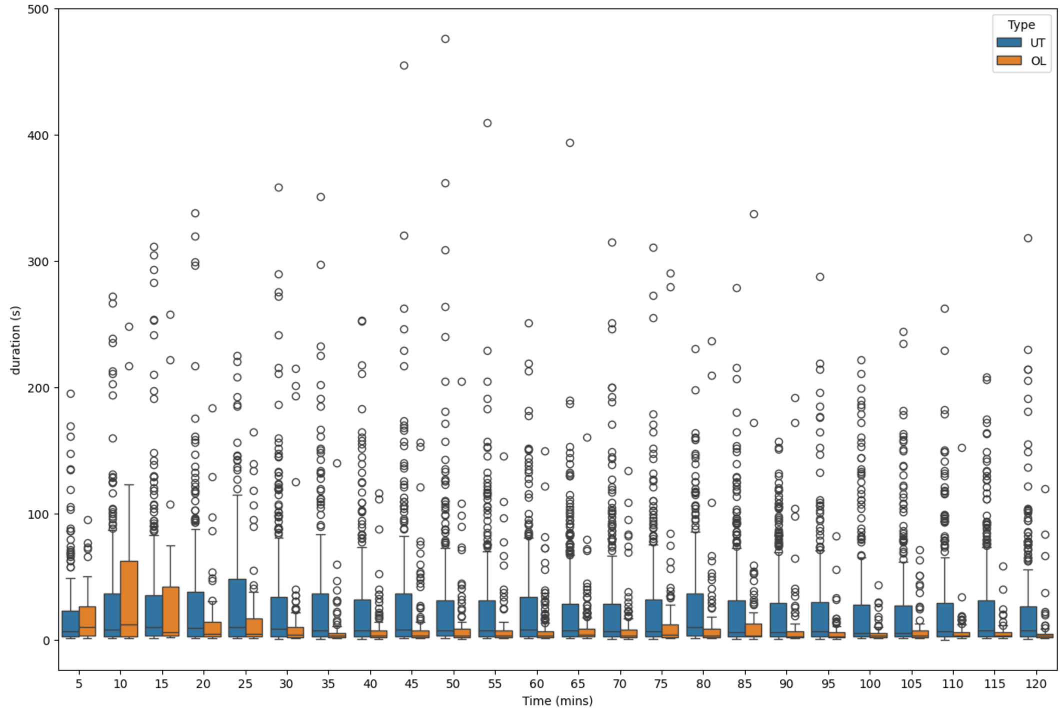

Supplementary Figure S11. Boxplots of track duration for the Banfora strain across the time of the experiment. Both UT and OL datasets are displayed for comparison.

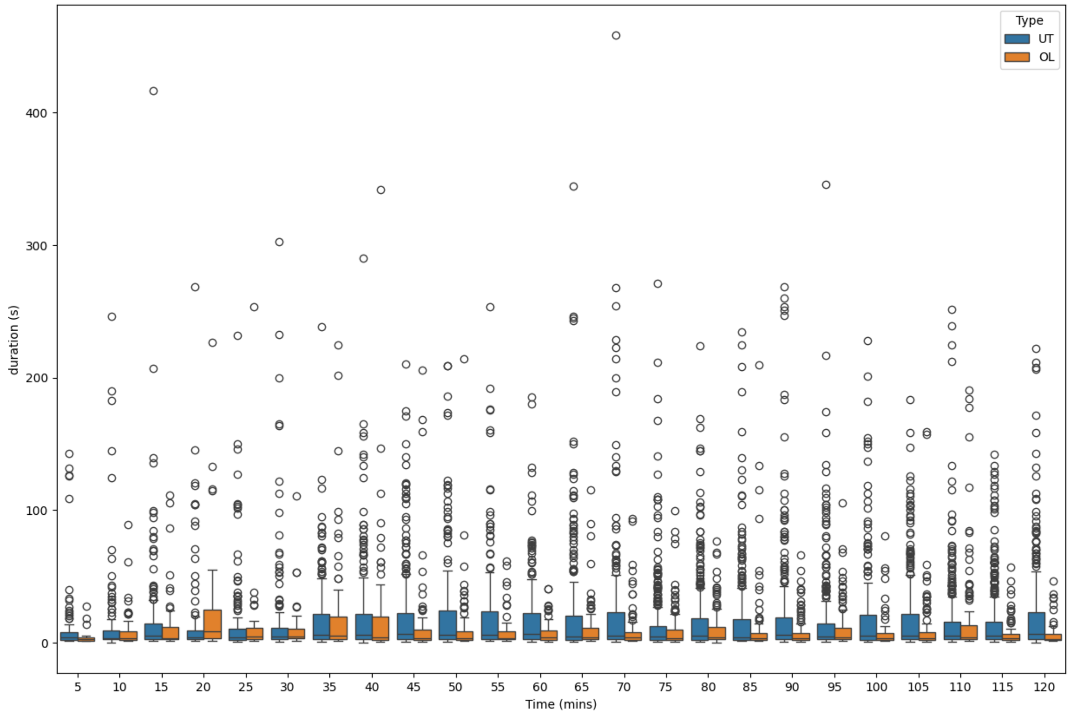

Supplementary Figure S12. Boxplots of track duration for the VK7 strain across the time of the experiment. Both UT and OL datasets are displayed for comparison.

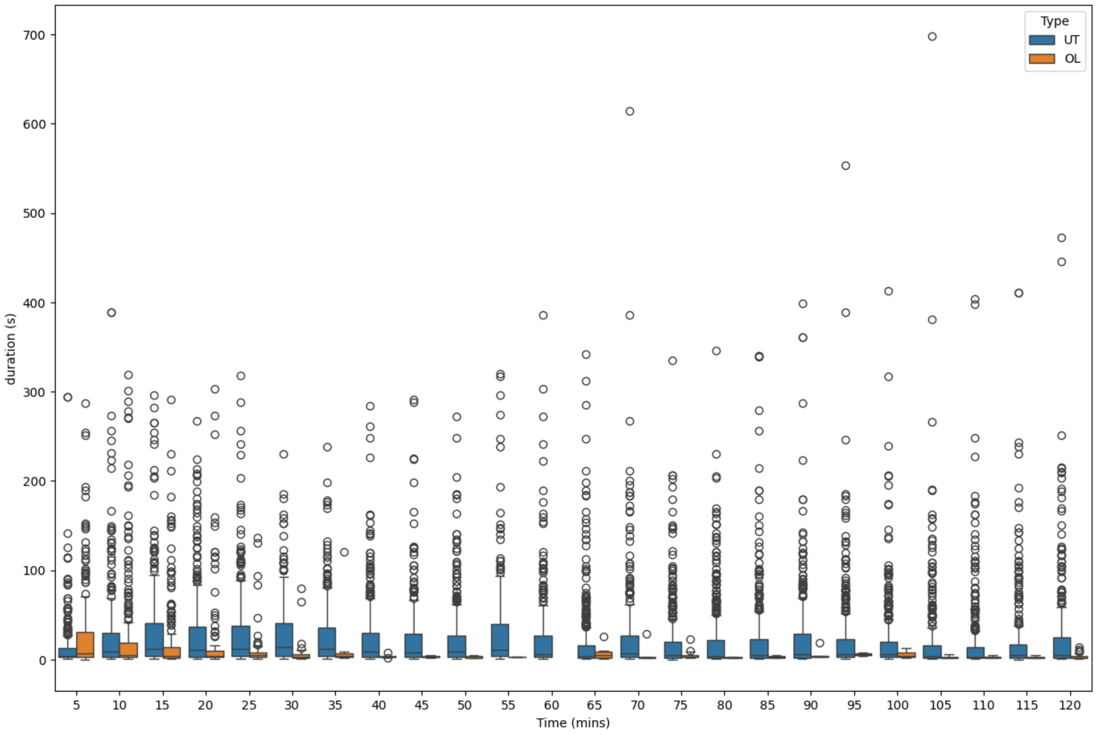

Supplementary Figure S13. Boxplots of track duration for the Kisumu strain across the time of the experiment. Both UT and OL datasets are displayed for comparison.

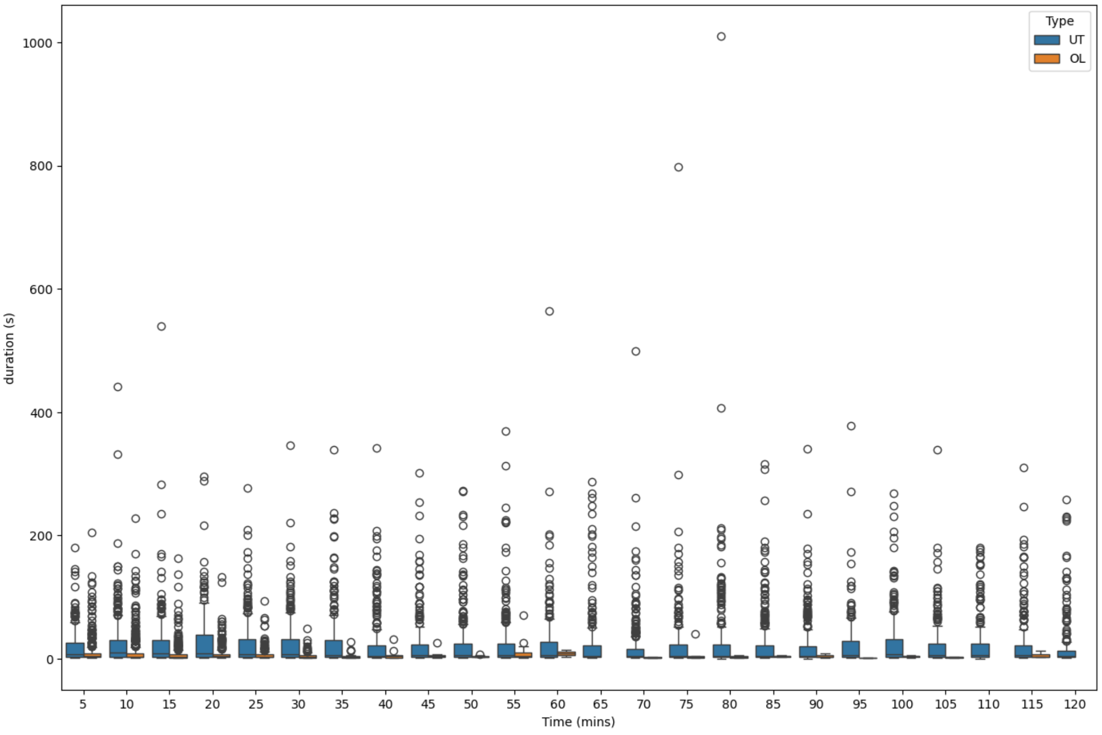

Supplementary Figure S14. Boxplots of track duration for the Ngoussu strain across the time of the experiment. Both UT and OL datasets are displayed for comparison.

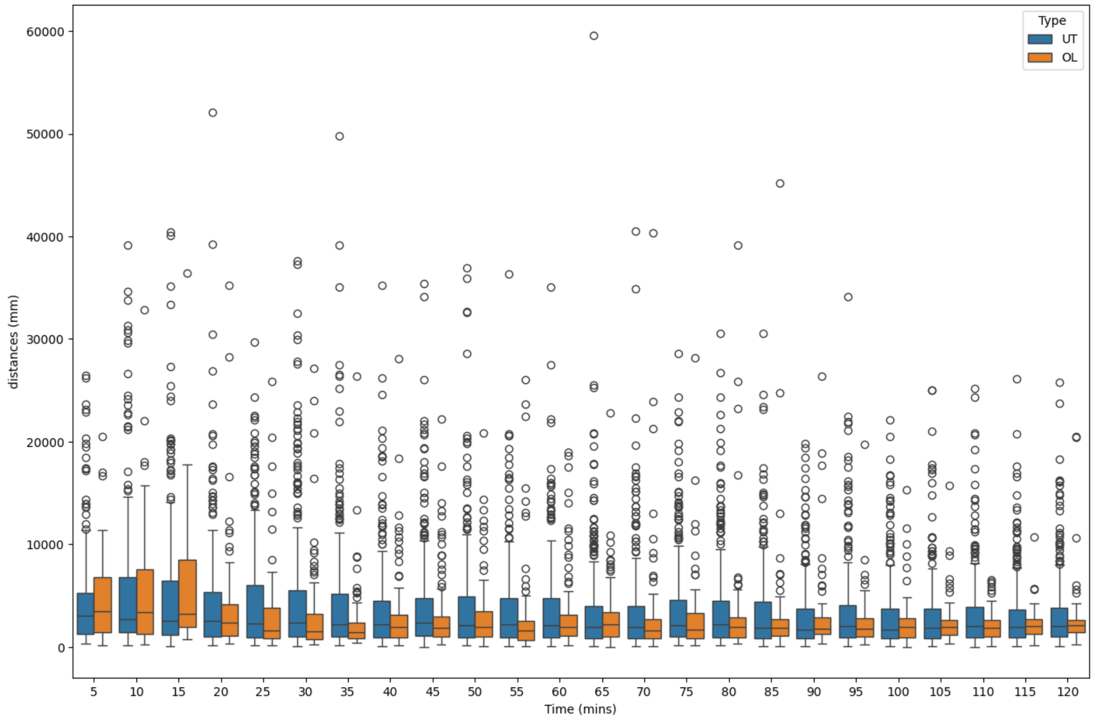

Supplementary Figure S15. Boxplots of track displacement for the Banfora strain across the time of the experiment. Both UT and OL datasets are displayed for comparison.

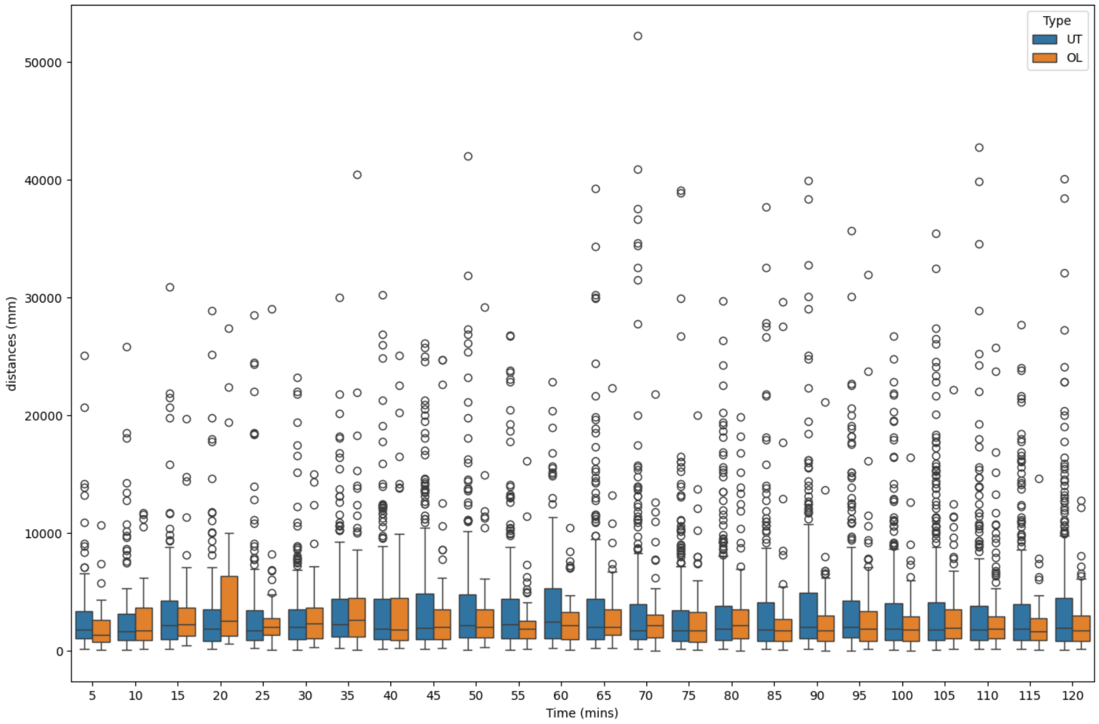

Supplementary Figure S16. Boxplots of track displacement for the VK7 strain across the time of the experiment. Both UT and OL datasets are displayed for comparison.

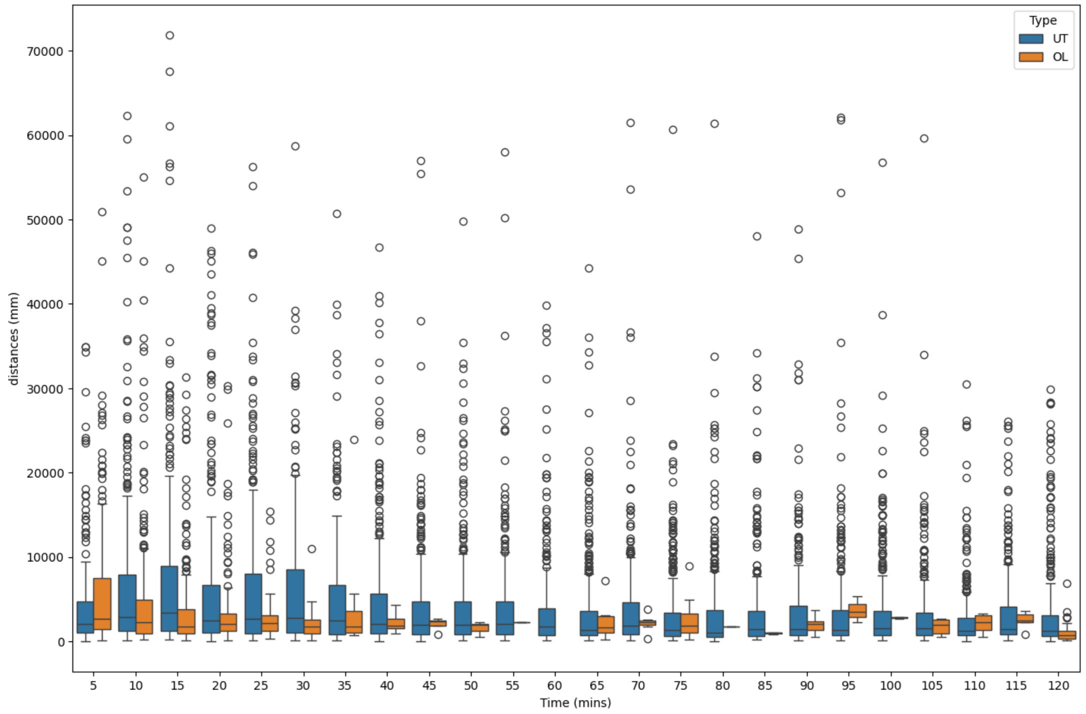

Supplementary Figure S17. Boxplots of track displacement for the Kisumu strain across the time of the experiment. Both UT and OL datasets are displayed for comparison.

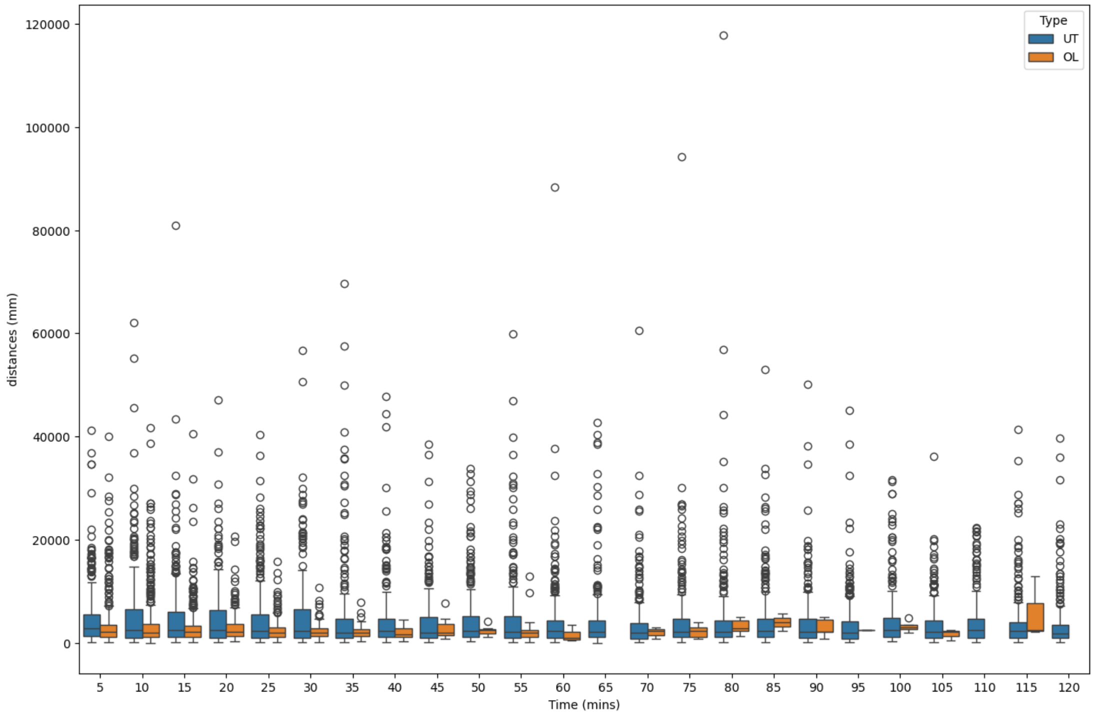

Supplementary Figure S18. Boxplots of track displacement for the Ngoussu strain across the time of the experiment. Both UT and OL datasets are displayed for comparison.

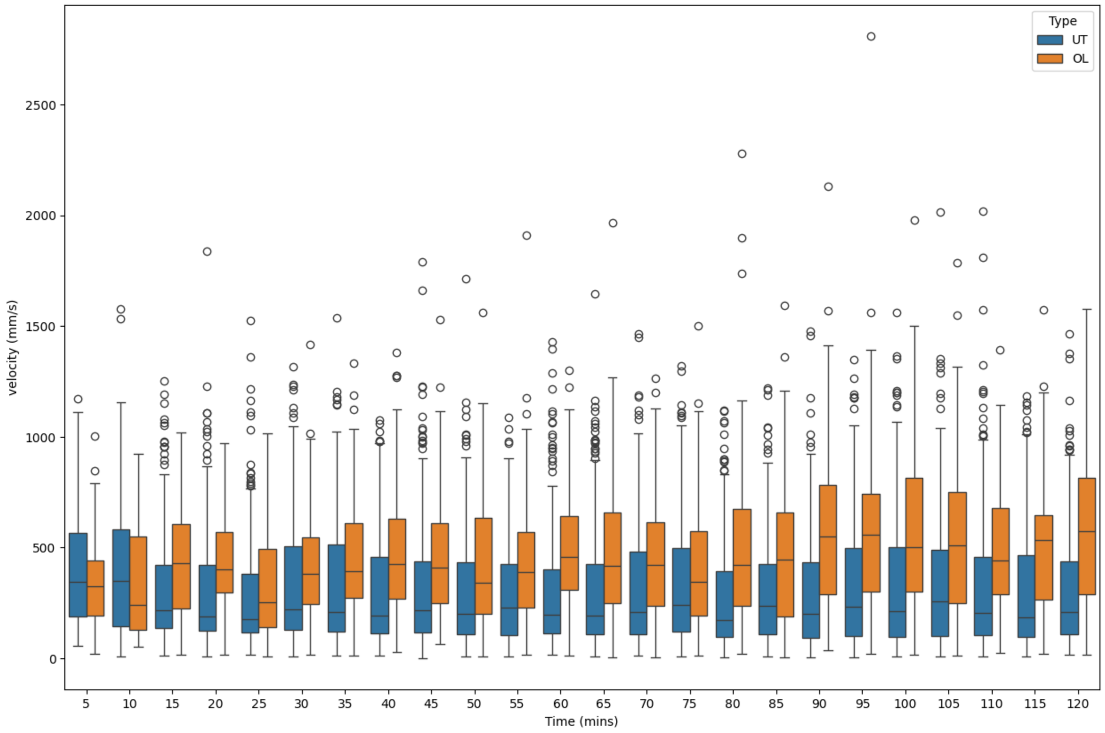

Supplementary Figure S19. Boxplots of average track velocity for the Banfora strain across the time of the experiment. Both UT and OL datasets are displayed for comparison.

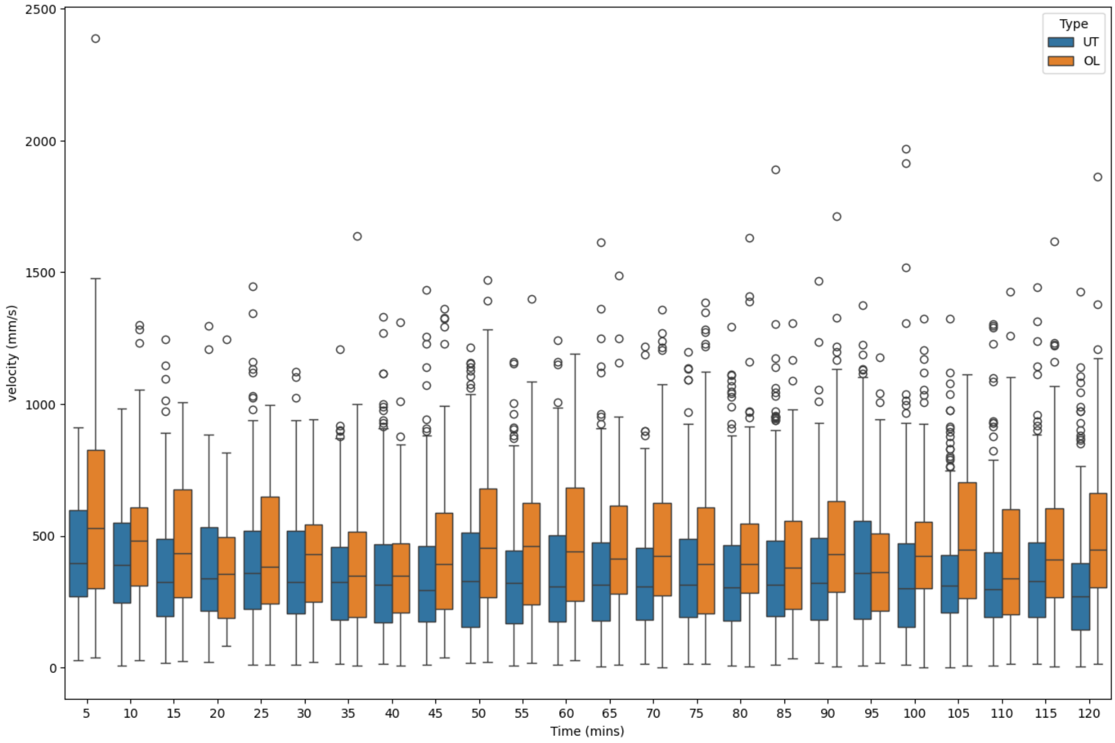

Supplementary Figure S20. Boxplots of average track velocity for the VK7 strain across the time of the experiment. Both UT and OL datasets are displayed for comparison.

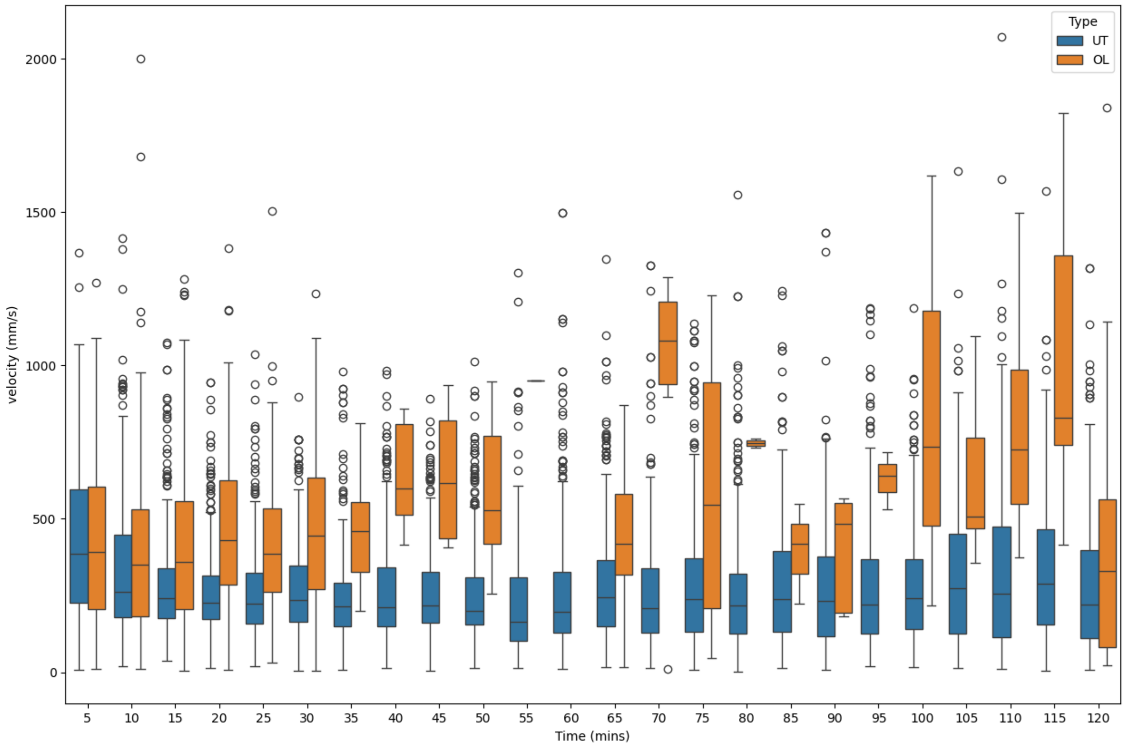

Supplementary Figure S21. Boxplots of average track velocity for the Kisumu strain across the time of the experiment. Both UT and OL datasets are displayed for comparison.

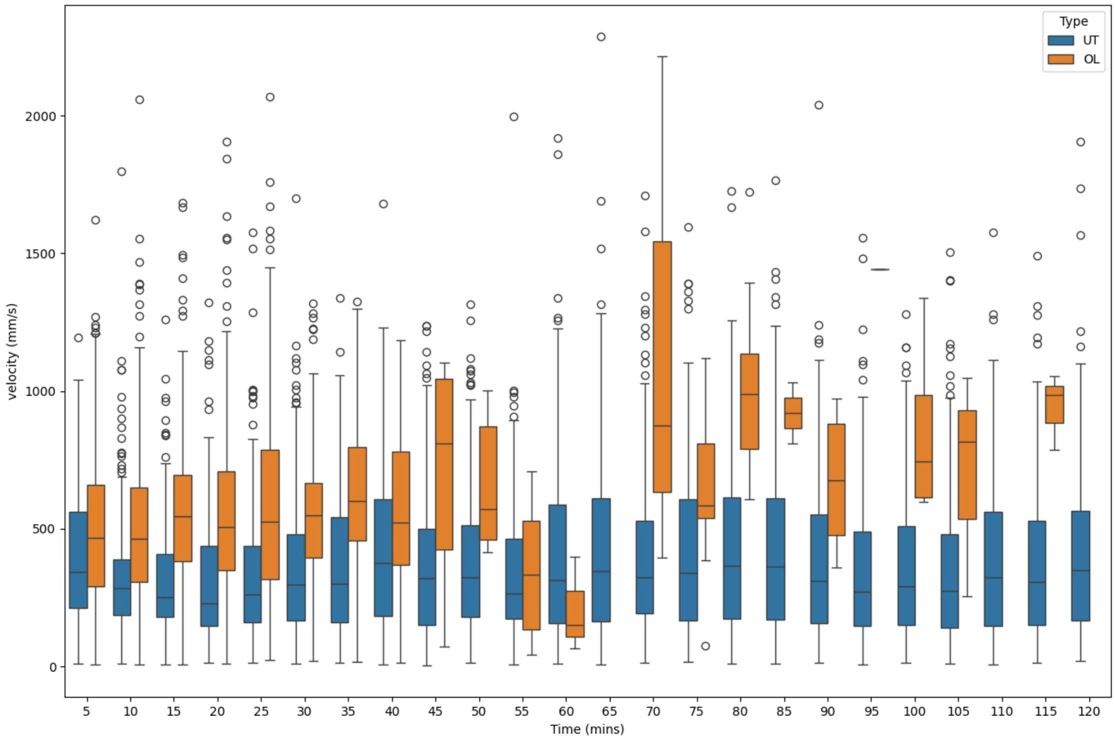

Supplementary Figure S22. Boxplots of average track velocity for the Ngoussu strain across the time of the experiment. Both UT and OL datasets are displayed for comparison.
